# Enhancement of Tumor Cell Immunogenicity and Antitumor Properties Derived from Platinum-Conjugated Iron Nanoparticles

**DOI:** 10.3390/cancers15123204

**Published:** 2023-06-15

**Authors:** Ángela-Patricia Hernández, Laura Iglesias-Anciones, José Javier Vaquero-González, Rafael Piñol, Julio J. Criado, Emilio Rodriguez, Pablo Juanes-Velasco, Marina L. García-Vaquero, Carlota Arias-Hidalgo, Alberto Orfao, Ángel Millán, Manuel Fuentes

**Affiliations:** 1 Department of Medicine and General Cytometry Service-Nucleus, CIBERONC CB16/12/00400, Cancer Research Centre (IBMCC/CSIC/USAL/IBSAL), IBSAL, University of Salamanca-CSIC, Campus Miguel de Unamuno, s/n, 37007 Salamanca, Spain; angytahg@usal.es (Á.-P.H.); l.anciones@usal.es (L.I.-A.); javiervaquero@usal.es (J.J.V.-G.); pablojuanesvelasco@usal.es (P.J.-V.); mlgarciavaquero@usal.es (M.L.G.-V.); carlotaariashidalgo@usal.es (C.A.-H.); orfao@usal.es (A.O.); 2 Department of Pharmaceutical Sciences, Organic Chemistry Section, Faculty of Pharmacy, University of Salamanca, Campus Miguel de Unamuno, s/n, 37007 Salamanca, Spain; 3 Institute of Nanoscience and Materials of Aragon (INMA), CSIC-University of Zaragoza, 50009 Zaragoza, Spain; pinol@unizar.es; 4 Department of Inorganic Chemistry, Faculty of Chemical Sciences, Plaza de los Caídos, s/n, 37008 Salamanca, Spainerodri@usal.es (E.R.); 5 Proteomics Unit, Cancer Research Centre (IBMCC/CSIC/USAL/IBSAL), 37007 Salamanca, Spain

**Keywords:** iron nanoparticles, cisplatin, antitumoral, immunogenic cell death, cytotoxicity, proteomics, microarrays

## Abstract

**Simple Summary:**

The immune system has been shown to play a critical role in controlling tumor development. Therefore, therapeutic strategies based on targeting tumors from this point of view are becoming increasingly important. One of the approaches offered by nanotechnology and small molecules in this context is the induction of immunogenic cell death (ICD). In this work, a characterization of the antitumor response of paramagnetic iron nanoparticles conjugated with a platinum compound is presented. A highlight of this work is the significant improvement in the antitumor properties of the nanoconjugate comparing it with the platinum compound. Moreover, the conjugation has been shown to have crucial properties for the induction of ICD, such as the activation of cellular endoplasmic reticulum stress. Our results emphasize the relevance of nanotechnology as a fundamental strategy for the development of new approaches with onco-immunotherapy activity.

**Abstract:**

From chemistry design to clinical application, several approaches have been developed to overcome platinum drawbacks in antitumoral therapies. An in-depth understanding of intracellular signaling may hold the key to the relationship of both conventional drugs and nanoparticles. Within these strategies, first, nanotechnology has become an essential tool in oncotherapy, improving biopharmaceutical properties and providing new immunomodulatory profiles to conventional drugs mediated by activation of endoplasmic reticulum (ER) stress. Secondly, functional proteomics techniques based on microarrays have proven to be a successful method for high throughput screening of proteins and profiling of biomolecule mechanisms of action. Here, we conducted a systematic characterization of the antitumor profile of a platinum compound conjugated with iron oxide nanoparticles (IONPs). As a result of the nano-conjugation, cytotoxic and proteomics profiles revealed a significant improvement in the antitumor properties of the starting material, providing selectivity in certain tumor cell lines tested. Moreover, cell death patterns associated with immunogenic cell death (ICD) response have also been identified when ER signaling pathways have been triggered. The evaluation in several tumor cell lines and the analysis by functional proteomics techniques have shown novel perspectives on the design of new cisplatin-derived conjugates, the high value of IONPs as drug delivery systems and ICD as a rewarding approach for targeted oncotherapy and onco-immunotherapies.

## 1. Introduction

Nanotechnology has emerged as a fundamental tool to improve the biopharmaceutical profiles of currently available drugs and small molecules as potential therapeutic drugs. These functionalization strategies have played a key role not only for novel drugs, but also for those conventional therapies that can be reintroduced or repurposed for different clinical applications [1]. A notable example is platinum derivatives and current clinical applications in combination with onco-immunotherapies [2]. Cisplatin and its most widespread derivatives (carboplatin and oxaliplatin) have presented hindered bioavailability by reduced distribution and delivery. They also presented a poor pharmacological profile by affecting renal function or by toxic accumulation in the organism [2]. Another significant obstacle to the development of platinum therapies is the emergence of resistance [3]. Chemical functionalization has achieved successful results in terms of solubility or drug profile but, in an attempt to overcome all the disadvantages in an effective manner, numerous nanotechnological strategies have been employed to increase the pharmacological potency and also minimize their adverse side effects. Several examples are collected in recent literature, including from nanogels, micelles, or liposomes to quantum dots, polymeric or inorganic-based nanoparticles (NPs) [4,5,6]. Beyond the classic effect of drug cytotoxicity, the immunomodulatory profile is becoming increasingly relevant in targeted oncotherapies. In this regard, it seems that nanotechnology has also played a key role in promoting onco-immunological activity [7].

Nanotechnology and platinum therapies have demonstrated to converge in their antitumor profiles in immunogenic cell death (ICD) [8]. Apart from the regulated cell death, a widely described mechanism in the action of many antitumoral therapies, ICD has been shown to be a rather widespread mechanism among cell death patterns of novel and also currently available drugs, including natural or synthetic entities. Moreover, ICD is combined with different antitumoral approaches such as monoclonal antibodies and radiotherapy [9]. The ICD involves initial endoplasmic reticulum (ER) stress, which promotes the activation of mitochondrial chaperones and the release of damage-associated molecular patterns (DAMPs), being exposed on the cell membrane or reaching the tumor microenvironment [10]. DAMPs such as calreticulin, HMGB1, ATP, or type I IFN have been described as hallmarks of ICD together with the ER stress [11]. DAMPs serve as an “alarm” for immune cells to identify the tumor cell and activate the immune response, thus modulating the tumor microenvironment in favor of tumor suppression. Thus, ICD induction has been described as a therapeutical approach but also as a prognostic factor for tumor progression, which is also considered as a potential biomarker in onco-immunotherapy [12,13].

Within the wide variety of available nanocomplexes, iron oxide nanoparticles (IONPs) present most of the essential characteristics that make them suitable for onco-immunotherapy. Classical applications of IONPs are related to diagnosis and therapy, such as magnetic resonance images (as contrast agents) or in hyperthermia [14,15,16,17]. Due to their chemical design including a surface polymer coating, these IONPs also allow conjugating different cytotoxic agents and in combination with other therapies they have been proved to display interesting immune properties [18,19]. The fruitful association of both features can be extensively exploited in drug development [20]. Even with the success of combining nanotechnology with several platinum complexes, there are still countless possibilities that can improve the association of both strategies. The conjugation of IONPs with bile acids functionalized cisplatin has proved to be a promising combination as the platinum cytotoxicity is enhanced [21], providing biocompatibility in biological systems [22] and allowing the multi-functionalization of IONPs at the same time [23]. In this study, a detailed analysis of cytotoxic and immune features of a novel bile-acid platinum complex conjugated with IONPs is accomplished. The emphasis of our research has been the potential of nanotechnology to improve the platinum properties. Several studies have been conducted in different cell lines of solid and hematological tumors to elucidate compound potency and selectivity. The profile of these IONPs as an efficient antitumoral drug delivery system and as ICD inducers has been tested using high-throughput protein microarray and analysis of both cell damage and immunogenic capacity.

## 2. Materials and Methods

### 2.1. Chemicals

Dimethyl sulfoxide (DMSO) was obtained from Merck (Darmstadt, Germany). Bovine serum albumin (BSA), trypan blue, urea, and 3-(4,5-dimethylthiazol-2-yl)-2,5-diphenyltetrazolium bromide (MTT) were purchased from Sigma (St Louis, MO, USA), and 96-well plates and BCA protein assay kit from Thermo Scientific (Rockford, IL, USA). Heat inactivated fetal bovine serum (FBS), L-glutamine, penicillin–streptomycin (P-S), and 0.25% trypsin-EDTA were purchased from Gibco^®^ (Gran Island, NY, USA), and 6-well clear flat bottom plates were purchased from Corning (Corning, NY, USA). Further details about the reagents of the chemistry section can be found in previous reports [17,23].

### 2.2. Chemistry

#### 2.2.1. Preparation and Characterization of Platinum Derivate

First, 122 mg (0.27 mmol) of glycoursodeoxycholic acid (HGUDCA) was added to water (200 mL) and sodium carbonate to facilitate dissolution of bile acid. The solution was then heated at 60 °C until completely dissolved. To this solution, another aqueous solution (200 mL) of [PtCl_2_(en)] (52 mg, 0.16 mmol) was slowly added dropwise. It was stirred in the dark at 60 °C for 24 h. Next, extraction with methanol was carried out using C-18 cartridges. The solvent was evaporated and the residue was extracted with 4 fractions of methanol (10 mL/fraction). The solution was then slowly applied to a preparative chromatography plate (TLC 60F254, 2 mm thick) using isobutyl acetate-methanol (35/65% *v*/*v*) as eluent. The compound was extracted with methanol, obtaining 19 mg (17%) of [PtCl(GUDCA)en]. Physical appearance: slightly yellowish solid. Elemental analysis of PtC_28_H_50_N_3_O_5_Cl (% calc./obt.): C, 45.49/44.85; H, 6.82/6.73; N, 5.68/5.60; Pt, 26.38/25.67. FT-IR (cm^−1^) (Supplementary File S1: Figures S1–S3): 3433.85 (νOH), 2928 (νCH), 2862. (νCH), 1632 (νasCOO), 1551 (νsCOO), 1411(CONH), 1348, 1048, 606. ^1^H-NMR (Supplementary File S1: Figures S4, S6 and S8) (ppm, CD_3_OD): 0.72 (3H, C18), 0.96 (3H, C19), 1.03 (3H, C21), 2.50 (4H, C1″, C2″), 4.03 (2H, C1′). ^13^C-NMR (Supplementary File S1: Figures S5 and S7) (ppm, CD_3_OD): 12.5 (C18), 19.88 (C19), 22.0 (C21), 24.1 (C19), 27.8 (C15), 49.2 (C1″, C2″), 180.1 (C24), 189.9 (C2′). ^195^Pt-NMR (Supplementary File S1: Figures S9 and S10) (ppm, CD_3_OD): −2269. 2D-NMR (Supplementary File S1: Figures S11–S15) M: Mass spectrum: 703.23 [M − Cl], 725.22 [M − Cl + Na] (Supplementary File S1: Figure S16).

#### 2.2.2. Synthesis of IONPs

**Figure 1:**
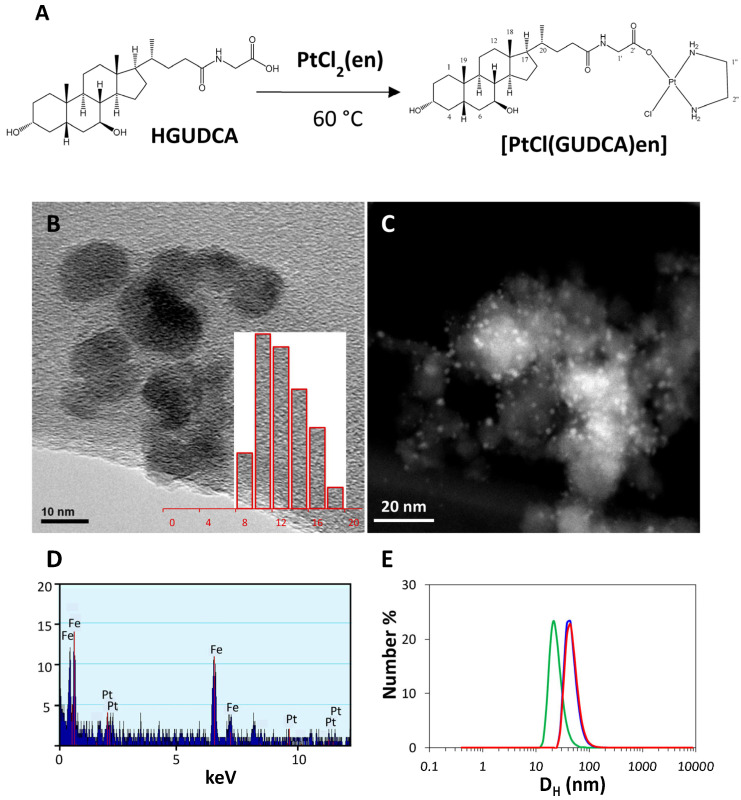
(**A**) Schematic representation of the preparation of platinum derivate [PtCl(GUDCA)en]; (**B**) TEM image and size distribution histogram of IONPs before coating and functionalization; (**C**) STEM image of the IONP@BCP@[Pt(GUDCA)_2_en] NPs; (**D**) EDS spectrum of IONP@BCP@[Pt(GUDCA)_2_en] NP sample; (**E**) Distribution of hydrodynamic diameters of NPs before coating (green), after coating (blue), and after functionalization with [Pt (GUDCA)_2_en complexes (pink).

Iron oxide magnetic stock nanoparticle (IONPs) suspension was prepared by alkaline coprecipitation of FeCl_3_·4H_2_O (83.2 g, 0.513 mol) and FeCl_2_·4H_2_O (51.4 g, 0.253 mol) salts dissolved in HCl (0.165 M, 400 mL) by dropwise addition of concentrated NH_4_OH at room temperature under mechanical stirring, until pH 9.2 was reached. The precipitate was separated by magnetic decantation, washed six times in water, and then it was treated with HNO_3_ 3 M for 2 h, filtered, redispersed by sonication in HCl 0.01 M, and filtered through 0.45 and 0.22 μm nitrocellulose filters (Millipore, Burlington, MA, USA). The final ɣ-Fe_2_O_3_ concentration in the suspension was 4 mg/mL as determined by ICP-OES. Synthesis of precursors rhodamine labeled fluorescent amphiphilic P4VP-b-P(MPEGA-co-RhodPEGMA-co-carboxylicPEGMA) block copolymer ((poly(4-vinylpyridine)-block-poly(methoxy poly(ethylenglycol) acrylate-co-Rhodamine poly(ethylenglycol) methacrylate-co-carboxylic poly(ethylenglycol) methacrylate, Mn: 16,500 Da) has been described in Ref. [16]. The block copolymer was further functionalized with carboxylic groups through post-polymerization modification of the terminal hydroxyl group of the pendant PEGMA chains with succinic anhydride. ^1^H-NMR spectrum of the final BCP polymer was consistent with the chemical structure in Figure 1. Polymer coating of iron oxide nanoparticles (IONP@BCP) was performed as follows: 37.5 mg of BCP were added to 15 mL of IONPs suspension at pH = 2. The copolymer is completely soluble at this pH, due to the protonation of pyridine groups in the P4VP block. As the pH is slowly increased by addition of NaOH 0.1 M, the pyridine groups are deprotonated and the P4VP block turns hydrophobic, encapsulating the nanoparticles. The suspension was sonicated, filtered through a 0.22 μm membrane filter, and purified by magnetic separation (MACS^®^ Miltenyi Biotec, Westphalia, Germany). The final volume was 15 mL, and he ɣ-Fe_2_O_3_ concentration determined by ICP-OES was 3.07 mg/mL.

#### 2.2.3. Incubation of IONPs with [PtCl(GUDCA)en]

The platinum complex with glycoursodeoxycholic acid [PtCl(GUDCA)en] was incubated with IONP@BCP NPs for 24 h, incubating a 0.097 mg/mL suspension in a 4.95 × 10^−4^ M solution of [PtCl(GUDCA)en], thus obtaining a suspension of IONP@BCP@[PtCl(GUDCA)in] NPs.

#### 2.2.4. Physical Characterization

Inductively Coupled Plasma Optical Emission Spectrometry (ICP-OES) was performed in a plasma 40 ICP Perkin-Elmer spectrometer. Samples for ICP-OES were prepared by freeze drying the SPIONs suspensions and digestion of the solid in concentrated HNO_3_ overnight. Dynamic light scattering (DLS) and zeta potential measurements of ferrofluids were performed using a Malvern Zetasizer NS (Malvern Instruments Ltd., Worcestershire, UK). The measurements were performed after dilution in ultra-pure water and were repeated at least three times on each sample to ensure consistency. Cryo Scanning Transmission Electron Microscopy (cryoSTEM) observations were carried out on a FEI Tecnai F30 microscope (FEI, Hillsboro, OR, USA).

#### 2.2.5. Release of the Platinum Complex Bound to the NPs

The incubated mixture of the IONPs with the platinum complex, IONP@BCP@[PtCl(GUDCA)en] (1 mL), was placed in an Eppendorf whose top had been replaced by a cellophane membrane (filter of >12,000 Da). The system was placed in contact with 5 mL of ultrapure water, keeping the device closed to prevent evaporation. It was observed that only the released platinum complex crosses the membrane. The absorbance at 240 nm was then recorded against time. The data fitted a first-order kinetics and a half-life of t1/2 = ln2/k = 266.6 h.

### 2.3. Biological Assays

All the human cell lines [Jurkat, T-cell leukemia (DSMZ ACC 282); Caco-2 (ATCC^®^ HTB-37™); HT-29 (ATCC^®^ HTB-38™); H460 (ATCC^®^ HTB-177); A549 (ATCC^®^ CCL-185)] were cultured at 37 °C in a humidified CO_2_ incubator (5% CO_2_) in complete RPMI media (Jurkat) or DMEM media (Caco-2, HT-29, H460 and A549) (both supplemented with 10% (*v*/*v*) FBS and 1% (*v*/*v*) P-S). When the cells reached 80% confluence, they were subcultured. For the adherent cells (Caco-2, HT-29, H460 and A549), 0.25% trypsin-EDTA was used to detach the cells. When necessary, the cells were counted using a Neubauer counting chamber and dyed with Trypan Blue.

#### 2.3.1. Cell Viability

Evaluation of cytotoxicity of Pt compound and NP conjugated with Pt was performed by MTT assay standard protocol as previously adapted in our group and reported in [24]. Furthermore, 1000 H460 and A549 cells, 2500 Caco-2 and HT-29 cells, and 10,000 Jurkat cells per well were seeded in triplicate in 96-well plates (Adherent cells were seeded 24 h before the assay to allow attaching). Cells were incubated with the corresponding stimuli (Pt or NPs) for 24 and 72 h at different dilutions (10 μM–0.1 μM). Each condition was assayed in triplicate, performing the experiment twice. Once these periods of time elapsed, the supernatant was replaced with fresh medium and 20 μL per well MTT (5 mg/mL) were added in darkness. After 4 h MTT incubation, the supernatant was removed and 200 μL DMSO per well were added to stop the reaction. Absorbance at 570 nm was determined by Gen5™ software (BioTek U.S., Winooski, VT, USA). Viability was correlated using control (cells without treatment) and vehicle (medium). To calculate the IC_50_, the viability values were plotted against the logarithm of the concentration. Data were fit to a linear regression. IC_50_ was the concentration value 50% of cell viability [25]. T-test was applied to determine the statistical significance between the IC_50_ values of both conditions.

#### 2.3.2. Protein Microarray Assays

Cells were plated in 6-well plates at a density of 300,000 cells per well and incubated for 24 h. Then, in adherent cell medium replaced with medium from the adherent cells and all the cell lines were treated with solutions of the drugs studied at 10 μM concentration. After the incubation time, the adherent cells were trypsinized and collected in tubes. Suspended cells were collected directly into tubes. All cell lines were centrifuged at 1200 rpm for 5 min. Once centrifuged, the supernatant was removed and the cell pellet was washed with PBS, centrifuged at 13,000 rpm for 3 min twice. The cell pellet was frozen at −80 °C until further use.

- Cell lysis, protein extraction, and biotin labelling

The cell pellet was thawed and 100 μL of lysis buffer was added to the cell pellet (NaCl 140 μM, EDTA 50 μM, glycerol al 10%, Tris-HCl pH = 8.20 μM) together with 200 μL of TCEP (10 mM), 20 μL of PMSF (100 mM), 20 μL of NaF (100 mM), 20 μL of orthovanadate (100 mM), 2 μL of β-glycerophosphate (1 M), and 40 μL of sodium pyrophosphate (125 mM). The cells were resuspended with the added buffer, the samples were kept 15 min on ice and centrifuged at 16,000 rpm for 15 min at 4 °C. The supernatant with total proteins was collected for successive assays. The BCA protein assay kit was used for protein quantification. Samples were diluted (1:10 *v*/*v*) in PBS and 25 μL of each sample was seeded in a 96-well plate. A colorimetric titration was performed by adding 200 μL of the kit dilution according to the manufacturer’s instructions. The plate was shaken for 5 min and then incubated for 30 min. The absorbance was determined at 562 nm and the protein concentration was quantified from a standard line constructed by incubation of serial dilutions of bovine serum albumin. For each condition, 100 μg of protein in final volume of 100 μL PBS were treated with 15 μL of a biotin solution (0.62 mg/mL) and incubated for 2 h at 4 °C. After this time, a 0.5 M Tris-HCl solution pH = 8.5 (50 μL) was added and the samples were stored at −20 °C until incubation on the arrays [26].

- Protein arrays processing, image acquisition, and data processing

The protein arrays used in this work were designed following previous reports [27] and functionalized and printed at the Functional Proteomics Service of the Cancer Research Center according to the procedures for the preparation of protein arrays as described previously [28,29,30]. Arrays were randomly selected from the available batch of arrays. In washing chambers, arrays were blocked for 1 h at room temperature and shaking. After the blocking, the arrays were washed 3 times with distilled water. Incubation was carried out following the procedure developed in [27,30]. The arrays were scanned with SensoSpot Fluorescence (Miltenyi Imaging GmbH, Radolfzell, Germany). The generated TIFF images were analyzed using GenePix Pro 6.0 software (Molecular Devices, San Jose, CA, USA). Parameters were set to quantify the intensity values of the Cy3 fluorochrome (λ = 532 nm). The fluorescence signal of the protein microarrays was corrected by subtracting the background signal and then transformed to Z-score as described [31]. For the selection of proteins to be analyzed in more detail in this work, a functional enrichment analysis of Gene Ontology (GO) terms was performed and signaling pathways involved in apoptosis (GO:0097190), cell cycle (GO:0031571), and RE stress (GO:0034976) were selected. Both functional enrichment and visualization of protein–protein interactions were performed on the String data base. Proteins and their codification are collected in Supplementary File S2. Each protein analyte was evaluated in 2 biological replicates each with 3 technical replicates. Negative fluorescence signal was transformed to zero. As expected, the signal distribution was not normal and zero-inflated (Supplementary File S3). Therefore, non-parametric Mann–Whitney test was used to compare the protein relative abundance between groups (experiment Pt or NP-Pt vs. Control). Standardized effect size (Ratio) was calculated by subtracting the mean log2 fluorescence in condition 1 or 2 and mean log2 fluorescence in control and then dividing by the overall standard deviation of experiment and control (Supplementary File S3).

## 3. Results and Discussion

### 3.1. Chemistry

#### Preparation and Characterization of the Platinum Compound with Glycoursodeoxycholic Acid, [PtCl(GUDCA)en]

The conjugation efficiency and biocompatibility of bile-acid cisplatin conjugates have been analyzed as previously reported, as well as their inclusion in nanosystems [22,23,32]. Based on this research, from glycoursodeoxycholic acid (GUDCA), platinum complex [PtCl(GUDCA)en] was synthesized and characterized (Figure 1). The structures were confirmed by spectroscopic techniques (Supplementary File S1: Figures S17–S22). The FT-IR spectrum showed the bands characteristic of bile acids, highlighting the band at 606 cm^−1^ characteristic of bile acids. Likewise, a single signal appeared in the ^195^Pt-NMR spectrum located at −2269 ppm, consistent with the platinum coordination environment in this compound. Additionally, the compound showed fluorescence in aqueous solution. The fluorescence spectrum showed an emission band that extends over a wide area of the spectrum. The UV spectrum showed a shoulder at 240 nm. Next, conjugation of [PtCl(GUDCA)en] with IONPs was confirmed by UV-visible and fluorescence spectra. The visible spectra showed the band at 562 nm corresponding to rhodamine and a charge transfer that hides the characteristic shoulder of the platinum complex. The fluorescence spectrum of the IONP@BCP@[PtCl(GUDCA)en] incubated with the platinum complex displayed a notable increase in fluorescence intensity in rhodamine emission with respect to the NPs without the platinum complex, assigned to FRET mechanism. The proximity of both fluorophores could be calculated by overlapping the emission band of [PtCl(GUDCA)en], which acts as a donor, and the absorption band of rhodamine, which would be the acceptor. This overlap allowed us to calculate the most probable distance (Föster radius) between both fluorophores, which in this case is Ro = 40.3 Å. Values of the Föster radius in the range 20–90 Å indicate an efficient energy transfer between fluorophores [33]. Liberation of platinum complex from IONP@BCP@[PtCl(GUDCA)en] was determined by UV, determining a first-order kinetics (Supplementary File S1: Figure S23). TEM images of the IONPs sample before coating are shown in Figure 1B. Individual NPs have a rounded shape and a size of 10.4 ± 2.1 nm (mean ± SD) and they are grouped in agglomerates with an average size of 23.3 ± 2.0 nm. Electron Diffraction (ED) patterns of the NPs were consistent with a spinel crystal structure. STEM images of IONP@BCP@[PtCl(GUDCA)en] nanoparticles sample (Figure 1C) show the presence of bright dots with a size of about 2 nm around the iron oxide nanoparticles that may correspond to high electron density Pt atoms of the [Pt(GUDCA)en] complexes. The Pt signal in the EDS spectrum was weak in comparison with Fe signal (Figure 1D). The distribution of NP hydrodynamic diameters and zeta potential of the uncoated IONPs, BDP coated, and Pt functionalized nanoparticle suspensions was determined by DLS, and it is represented in Figure 1E as a function of the number frequency. A similar plot as a function of intensity frequency is shown in Supplementary File S1: Figure S24. The corresponding parameters are presented in Table 1 and Table S1. According to TEM and DLS diameters, and assuming a perfect spherical shape, the volume and surface areas of the final IONP@BCP@[PtCl(GUDCA)en] beads would be V = 5.76·10^−23^ m^3^ and S = 6.78·10^−15^ m^2^, respectively.

### 3.2. Biological Assays

#### 3.2.1. Tumor Cell Cytotoxicity by Viability Assays

**Figure 2:**
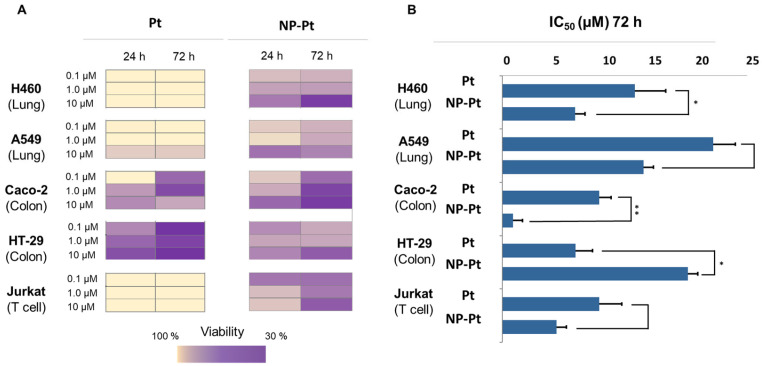
(**A**) Viability results (%) represented in a heatmap, comparing five different tumor cell lines (H460, A549, Caco-2, HT-29 and Jurkat), two times of drug exposure to the cells (24 and 72 h), and the two compounds evaluated, Pt complex (Pt) and NPs conjugated with Pt complex (NP-Pt); (**B**) Bar diagrams representing IC_50_ (μM) of Pt and NP-Pt at 72 h of incubation. Results are reported as the mean ± SD of IC_50_ values of three independent experiments (significantly differences * *p* < 0.05, ** *p* < 0.01).

In order to evaluate the tumor inhibition activity, [Pt(GUDCA)en] (named in this section as Pt) and novel IONP@BCP@[Pt(GUDCA)en] (named in this section as NP-Pt) were tested in several tumor cell lines. Viability was assessed in five different tumor cells: lung cancer cell lines (H460 and A549), colon carcinoma cell lines (Caco-2 and HT-29), and a T lymphocyte cell line (Jurkat). The time of incubation of the compounds with cells was also taken into consideration (24 h and 72 h) as well as drug concentration (0.1 μM, 1 μM, and 10 μM) to define an overall perspective of the activity of the platinum compound on its own and after conjugation to the NP (Figure 2 and Supplementary File S1: Figure S24). At first glance, several insights are observed in Figure 2. In view of the results, the Pt compound only induces cytotoxicity in the colon tumor cells Caco-2 and HT29 while the other cells evaluated were not sensitive to Pt. In these colon cell lines, the cytotoxicity increased as the incubation time of the cells with Pt increased. More interesting and heterogeneous are the results for NP-Pt. In lung tumor cells, when NP-Pt was tested, cytotoxicity was found at both incubation times (at 24 h and 72 h). In H460 cells, a progressive effect was observed over time, as NP-Pt is generally more cytotoxic at a longer incubation time and high concentration. In the A549 cell line at 72 h incubation, the viability observed is slightly different between conditions. In Caco-2 cells, when treated with NP-Pt, a time-concentration dependent response was observed. In the HT-29 cell line, where Pt had reached the best cytotoxicity values, it was observed that NP-Pt was not more cytotoxic, displaying a decrease in the response. When NP-Pt was tested on the Jurkat (hematological cell line), cytotoxicity was observed from the shortest incubation time, highlighting the value obtained at the lowest dilution (0.1 μM), remaining or increasing at 72 h of incubation.

Here, the focus was on the effect analysis of the cytotoxicity of the conjugate with respect to Pt. For this purpose, the differences between the IC_50_ values between NP-Pt and Pt were studied. As displayed in Figure 2B, all cell lines, except HT-29, presented a decrease in the IC_50_ value in the NP-Pt treated condition. It was detected that H460 cells showed a significant increase in cytotoxicity for NP-Pt with respect to the Pt. There is also a considerable increase, although not significant, in cytotoxicity in A549 and Jurkat cells between NP-Pt and Pt. However, the most interesting ratio is reached by NP-Pt in the Caco-2 line, where the IC_50_ value of NP-Pt is decreased up to nine-fold with respect to the reported IC_50_ of the platinum compound. These results suggest that the properties of the Pt have been improved in certain tumor cell lines by conjugation with NPs, and provide promising results for further research on signaling networks mediated by NP-Pt and Pt and their comparison.

#### 3.2.2. Analysis of Signaling Pathways by Antibody Microarrays

**Figure 3:**
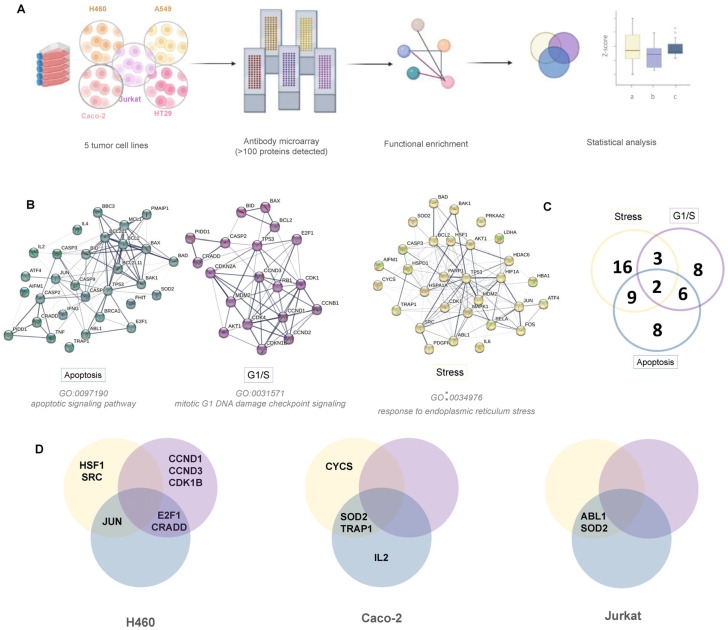
(**A**) Workflow of the evaluation assays and data analysis ((a) for control, (b) for Pt and (c) for NP-Pt); (**B**) Functional enrichment of proteins of interest in relation with the signaling pathways: stress, apoptosis, and cell cycle G1/S; (**C**) Venn diagram with the significative proteins determined for those signaling pathways; (**A**,**D**) Venn diagrams of the specific significative proteins for the corresponding tumor cell line.

Evaluation of the intracellular pathways has been carried out by incubation of biotin labeled cell lysates on antibody microarrays as depicted in Figure 3. For the statistical analysis, a functional enrichment was performed using the String platform to select the proteins of interest (Figure 3A and Supplementary File S2). The proteins analyzed were related to the pharmacology and immunomodulatory mechanism linked to platinum compounds.

**Figure 4:**
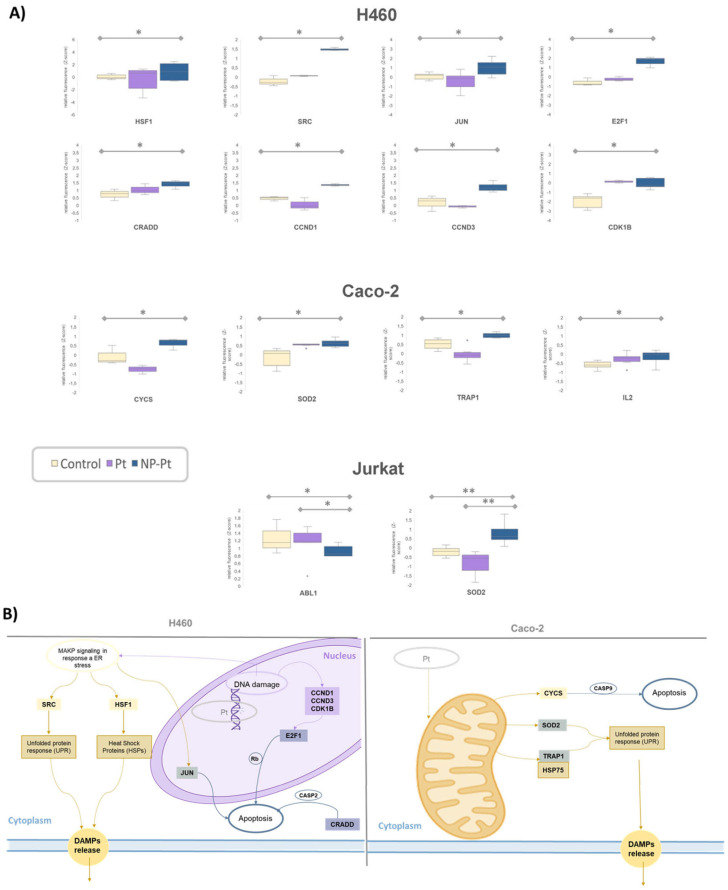
(**A**) Box plots representing the Z-score values obtained for studied proteins. Only proteins with significant differences in at least one comparison with control (* = *p* < 0.05; ** = *p* < 0.01) are depicted; (**B**) Schematic representation of ER stress- and ICD-related triggered intracellular signaling in H460 and Caco-2 cell line.

On one side, apoptosis triggered by DNA in the transition of G1/S phase of the cell cycle, in the DNA replication phase. On the other side, proteins involved in ER stress, a key feature for the development of ICD, have been analyzed (Figure 3B). Intracellular signaling analysis is complex and these processes are closely related within the context of cell death. Figure 3B shows the interconnection of proteins of each pathway as well as a Venn diagram (Figure 3C) shows the distribution of the proteins in each pathway and the number of common proteins between two of the three signaling pathways studied. At first glance, it was observed that only in the H460, Caco-2 and Jurkat cells obtained significant differences in protein activation when treated with Pt and NP-Pt. Considering the proteins according to intracellular cell signaling, it seems that the H460 cells are the ones where variations in the three pathways were detected. In the other two cell types, only variations in cell stress and apoptosis signaling were detected (Figure 4).

Subsequently, a more exhaustive analysis of the variations in intracellular protein levels concerning the Pt compound and the NP was conducted. It should be noted that the cells with significant variations were those in which the NP-Pt conjugate promoted greater cytotoxicity. These viability results can be correlated with the variation of intracellular protein concentrations (Figure 3D). Analyzing each cell line in detail (Figure 4A), it can be observed that in H460 cells, significant differences were detected between control cells and cells treated with NP-Pt. In most cases, protein variation follows the same trend between Pt and NP-Pt, although a significant difference was detected in the case of the conjugate (HSF1, SRC, E2F1, CRADD, and CCK1B). However, proteins have also been found where the Pt compound alone has not shown the same trend as the NP conjugate (JUN, CCND1, and CCND3).

In Caco-2 cells, there was a smaller number of significant proteins. In this case, proteins behaved similarly in the NP-Pt and in the Pt compound (SOD2 and IL2) and others that presented an opposite trend (TRAP1 and SOD2). Finally, in Jurkat cells, differences were only observed in two proteins, ABL1 and SOD2. In this case, the SOD2 protein was common to the Caco-2 line and presented different levels in Pt and NP-Pt.

This new bile-acid platinum complex and novel nanoconjugate demonstrated cytotoxic properties and immunomodulatory potential profile when testing in different tumor cell lines. The viability of cells was analyzed in different tumor cell lines, demonstrating changes in the behavior of the platinum complex and the nanoconjugate between them. This fact highlights the need to always explore several pharmacological targets to prevent toxicity and to establish a structure–activity relationship of the new compounds synthesized.

High-throughput functional proteomics analysis provided a correlation between intracellular signaling, cytotoxicity, and ICD patterns mediated by ER stress activation (Figure 4A). In the case of H460 cells, several proteins related to MAPK/ERK pathway signaling were activated: HSF1, SRC, and JUN. This signaling pathway is closely related to the ER stress response as well as to the response to DNA damage [34].

HSF1 and SRC proteins play a crucial role in the activation of ER stress and ICD. On the one hand, HSF1 is a transcription factor for the synthesis of “heat shock proteins” such as HSP90 or HSP70, chaperone-like proteins synthesized by the mitochondria in situations of cellular stress, and which are considered DAMPs when released in the cell death process [35]. Along the same lines, SRC, activated in stress situations, activates the unfolded protein response (UPR) stress cascade through the IRE1 pathway [36]. In addition, it has been possible, in these cells, to detect the damage to the genetic material by DNA alkylation related to platinum-specific activity. The activation of cell cycle control mechanisms in response to damage is reflected by CCND1, CCND3, and CDK1B. These cyclins trigger apoptosis mechanisms mediated by E2F1 activation. In this case, activation of apoptosis mediated by JUN (end effector in the MAPK/ERK pathway) and by CRADD (in response to ER stress) was also detected [37].

A similar insight was observed in the Caco-2 cells by activation of mitochondrial signaling. This organelle plays its role in the management of reactive oxygen species through the activation of proteins such as SOD2. This protein converts ROS into non-reactive substances and detoxifies the cell through biochemical proton/electron exchange reactions. In this case, the SOD2 protein is also described as participating in the activation of the UPR [38]. TRAP1, also known as HSP75, as the other related chaperones, is known to be closely related to ER stress and the ICD response [39]. In both cases, even though the downstream signaling pathways seem different, both tumor cell lines, H460 and Caco-2, showed protein cascades converging in the ER stress activation and the potential development of ICD response.

## 4. Conclusions

Nanoconjugates from platinum derivates and IONPs antitumoral properties have proved to be very interesting and selective in different tumor cell lines. The improvement of the properties of the platinum complex after inclusion in the nanosystem enhanced the activity significantly. In addition, promising immunogenic properties of the nanoconjugate have been described by ER stress activation. Thus, we can conclude that cisplatin-derived agents together with the high value of IONPs as drug delivery systems and immunogenic cell death are a promising approach to continue searching for novel strategies in nanotechnology and in antitumoral- and immunotherapy. High-throughput functional proteomics-based techniques also provide a suitable approach to deciphering intracellular pathways and slight differences in biopharmaceutical approaches.

## Figures and Tables

**Table 1:** Mean hydrodynamic diameter (DH), standard deviation (SD), polydispersity index (PDI), and Z-potential (Z) of the nanoparticle suspensions obtained from dynamic light scattering (DLS) measurements.

Sample	DH (nm)	SD (nm)	PDI	*z* (mV)
IONP	24.2	7.5	0.18	+40
IONP@BCP	46.5	14.2	0.14	−30
IONP@BCP@[PtCl(GUDCA)en]	47.9	14.8	0.22	−9

## Data Availability

The data presented in this study are available in this article (and Supplementary Materials).

## Supplementary Materials

The following supporting information can be downloaded at: https://www.mdpi.com/article/10.3390/cancers15123204/s1, Supplementary File S1: contains the complete chemical characterization of the reported compounds; Supplementary File S2: contains proteins and their description for intracellular pathways analyzed; Supplementary File S3: contains quality control of microarrays development and analysis.

## Abbreviations

DAMPs: Damage-associated molecular patterns; DH, Hydrodynamic diameter; DLS, Dynamic light scattering; ED, Electron Diffraction; ER, Endoplasmatic reticulum; GO, Gene Ontology; GUDCA, Glycoursodeoxycholic acid; ICD, Immunogenic Cell Death; IONPs, Iron oxide nanoparticles; MTT, 3-(4,5-dimethylthiazol-2-yl)-2,5-diphenyltetrazolium bromide; NMR, Nueclar Magnetic Resonance; NPs, Nanoparticles; PDI, Polydispersity index; P-S, Penicilin-Streptomicin; SD, Standard deviation; TEM, Transmission electron microscopy.
