# Supplementary material for: Enhancement of Tumor Cell Immunogenicity and Antitumor Properties Derived from Platinum-Conjugated Iron Nanoparticles
Source: Cancers (Basel). 2023 Jun 15;15(12):3204. doi: 10.3390/cancers15123204 (PMC10296818; doi:10.3390/cancers15123204)
Supplement: Supplementary file 1 [file cancers-15-03204-s001.zip › HernandezAP_Cancers_SuppFile1 new.pdf]

# Enhancement of tumor cell immunogenicity and antitumoral properties of a novel platinum derivative conjugated to iron nanoparticles.

Ángela-Patricia Hernández[a,b], Laura Iglesias-Anciones[a], José Javier Vaquero-González[a], Rafael Piñol[c], Julio J. Criado[d], Emilio Rodriguez[d], Marina L. García-Vaquero[a], Pablo Juanes-Velasco[a], Carlota Arias-Hidalgo[a], Alberto Orfao[a], Angel Millán[c*] and Manuel Fuentes[a,e*]

[a.] *Department of Medicine and General Cytometry Service-Nucleus, CIBERONC CB16/12/00400, Cancer Research Centre, (IBMCC/CSIC/USAL/IBSAL), University of Salamanca-CSIC, IBSAL, Campus Miguel de Unamuno s/n, 37007 Salamanca.*
[b.] *Department of Pharmaceutical Sciences. Organic Chemistry Section. Faculty of Pharmacy, University of Salamanca, Campus Miguel de Unamuno s/n, 37007 Salamanca, Spain*
[c.] *INMA, Institute of Nanoscience and Materials of Aragon, CSIC-University of Zaragoza, 50018 Saragossa, Spain*
[d.] *Department of Inorganic Chemistry, Faculty of Chemical Sciences, Plaza de los Caídos S/N, 37008 Salamanca, Spain.*
[e.] *Proteomics Unit, Cancer Research Centre (IBMCC/CSIC/USAL/IBSAL), 37007 Salamanca, Spain.*

# Supplementary Figures

## Chemical Characterization

Figure S1

Ptgudca1

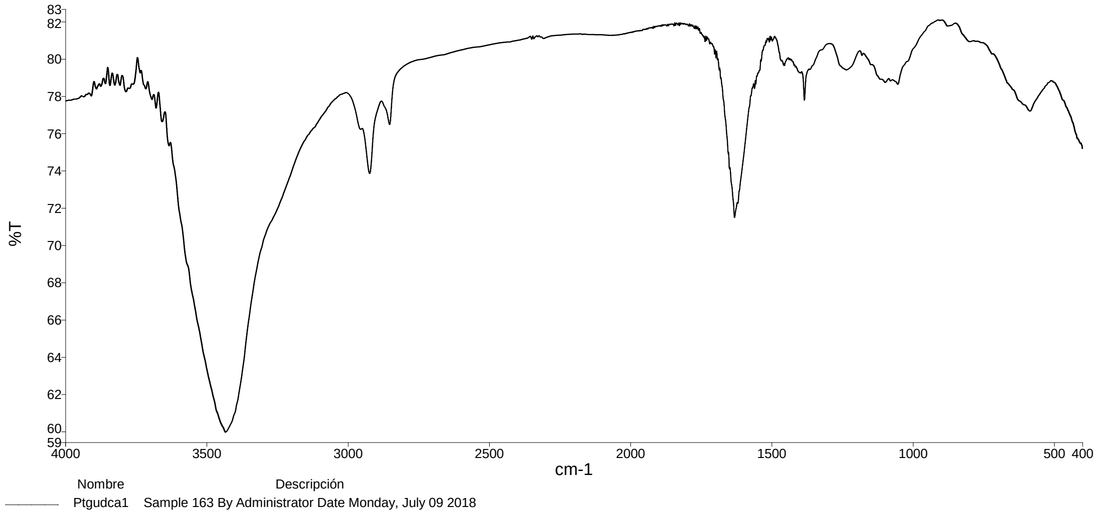


| Peak Number | X (cm-1) | Y (%T) |
|---|---|---|
| 1 | 3434.62 | 59.98 |
| 2 | 2924.27 | 73.88 |
| 3 | 1632.1 | 71.51 |
| 4 | 1384.47 | 77.81 |
| 5 | 1053.55 | 78.67 |
| 6 | 585.54 | 77.23 |

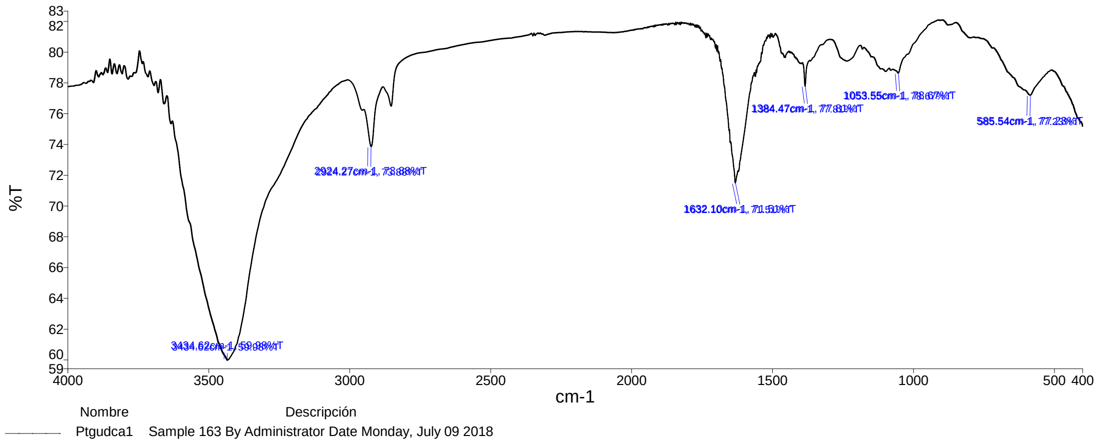

Figure S2

## Gudca1

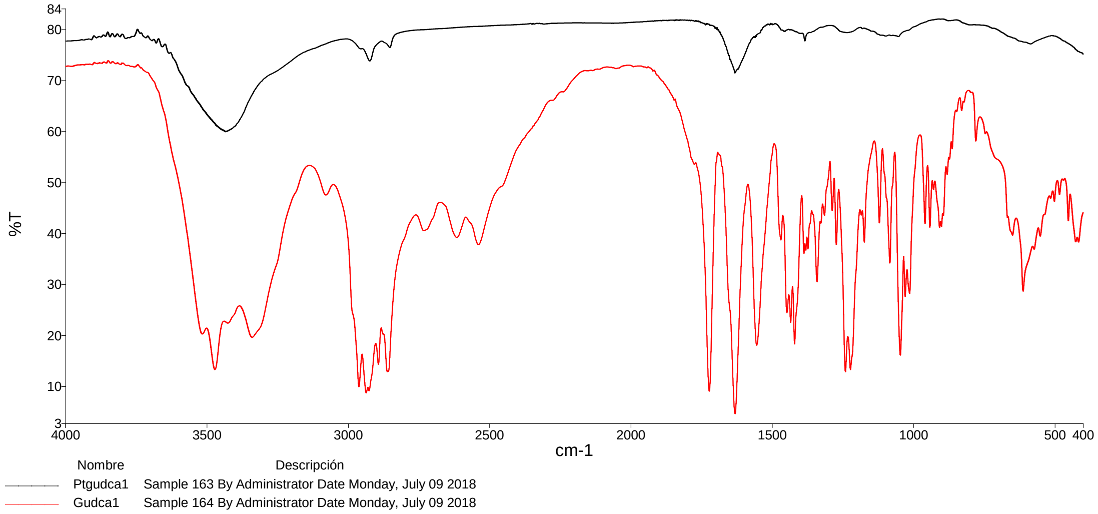


| Peak Number | X (cm-1) | Y (%T) |
|---|---|---|
| 1 | 2962.82 | 9.98 |
| 2 | 2937.06 | 8.77 |
| 3 | 2926.79 | 9.18 |
| 4 | 2861.99 | 12.94 |
| 5 | 1722.96 | 9.11 |
| 6 | 1631.83 | 4.69 |
| 7 | 1241.13 | 12.9 |

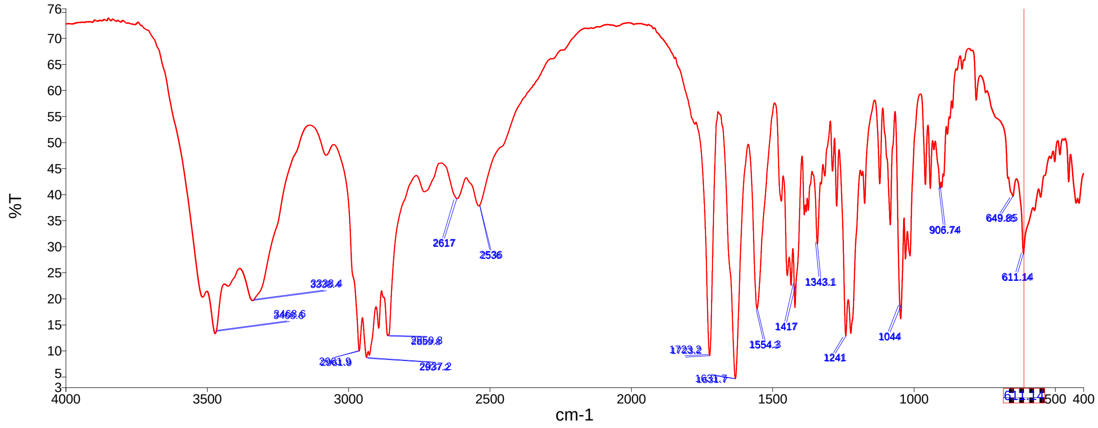

Figure S3

Ptgudca2

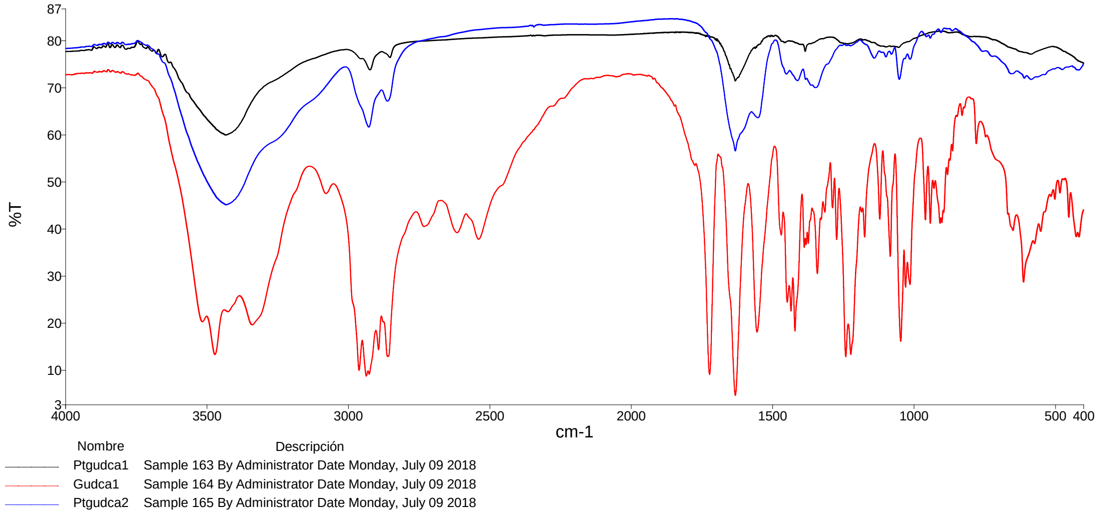


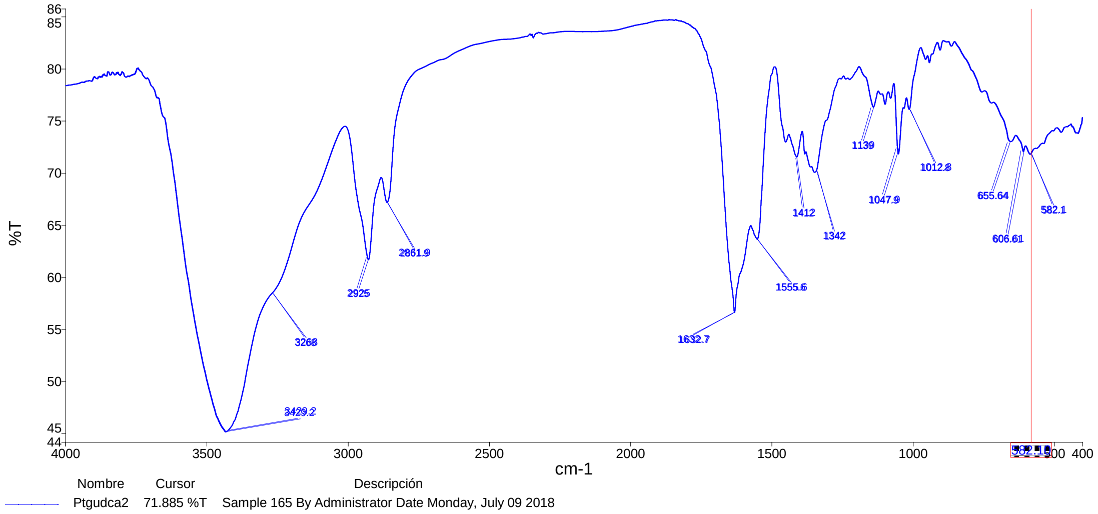


| Peak Number | X (cm-1) | Y (%T) |
|---|---|---|
| 1 | 3433.85 | 45.17 |
| 2 | 2928.44 | 61.69 |
| 3 | 2862.61 | 67.21 |
| 4 | 1631.99 | 56.62 |
| 5 | 1551.63 | 63.67 |

Figure S4

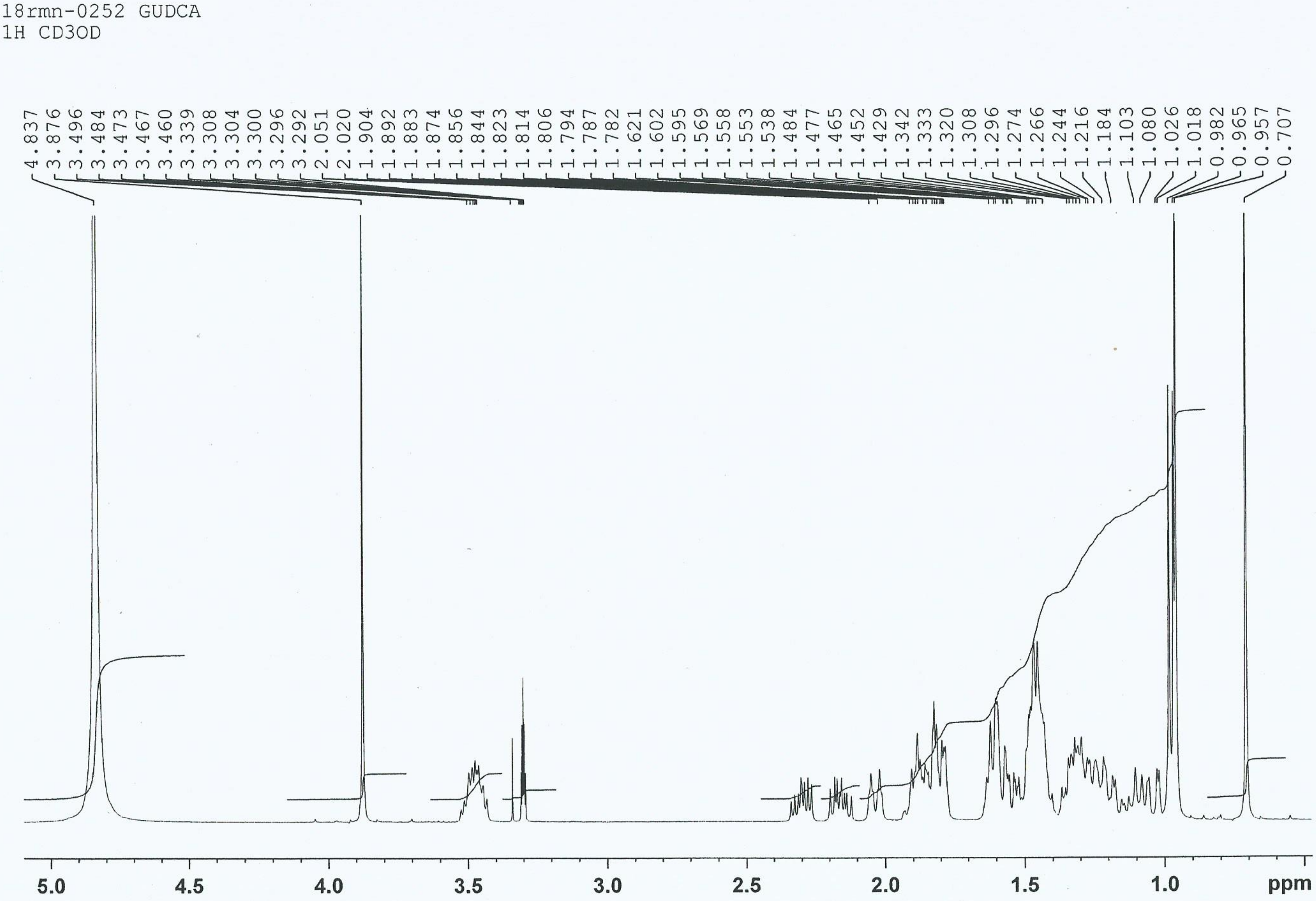
18rmn-0252 GUDCA
1H CD3OD
4.837
3.876
3.496
3.484
3.473
3.467
3.460
3.339
3.308
3.304
3.300
3.296
3.292
2.051
2.020
1.904
1.892
1.883
1.874
1.856
1.844
1.823
1.814
1.806
1.794
1.787
1.782
1.621
1.602
1.595
1.569
1.558
1.553
1.538
1.484
1.477
1.465
1.452
1.429
1.342
1.333
1.320
1.308
1.296
1.274
1.266
1.244
1.216
1.184
1.103
1.080
1.026
1.018
0.982
0.965
0.957
0.707
5.0
4.5
4.0
3.5
3.0
2.5
2.0
1.5
1.0
ppm

Figure S5

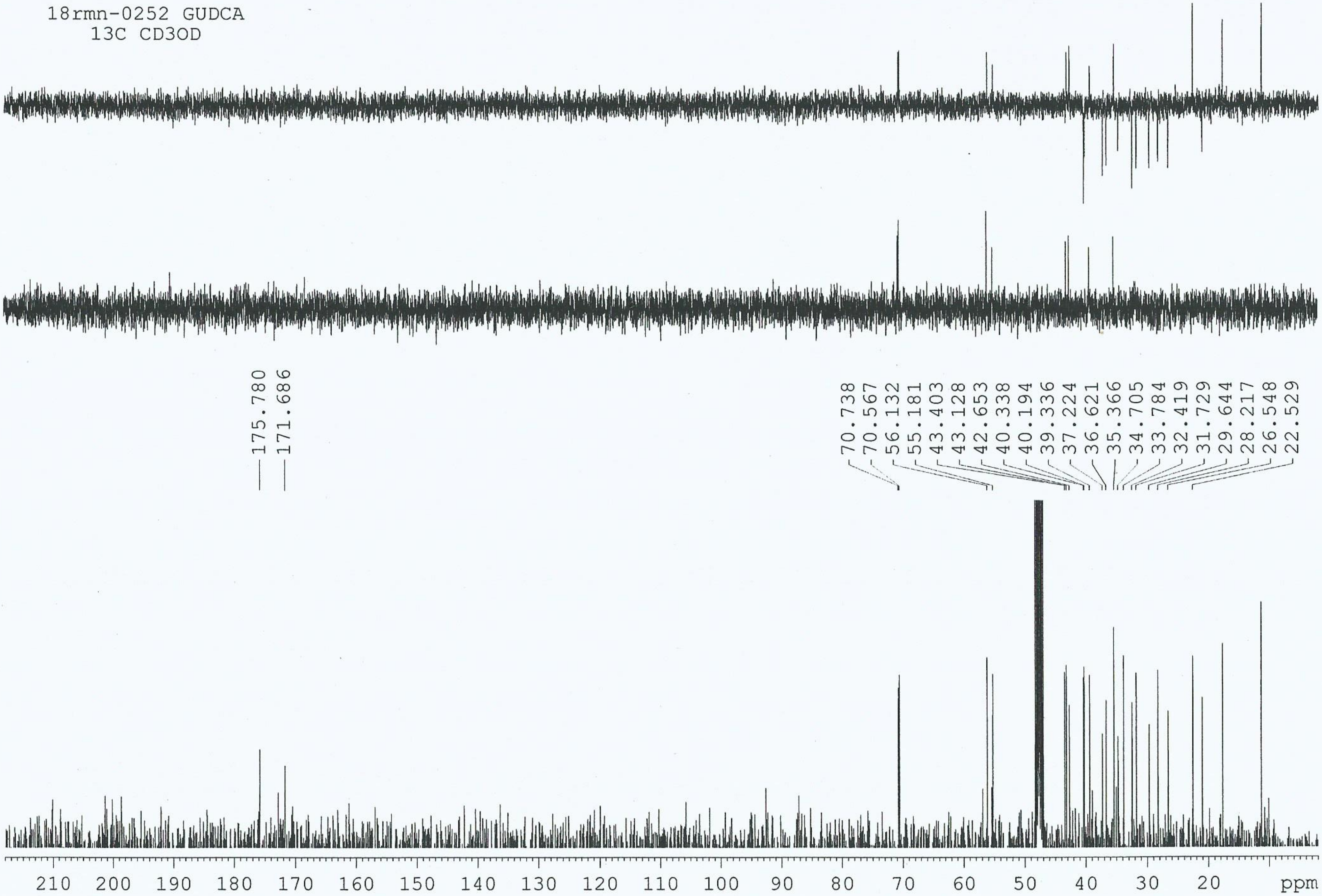

18rmn-0252 GUDCA
13C CD3OD
175.780
171.686
70.738
70.567
56.132
55.181
43.403
43.128
42.653
40.338
40.194
39.336
37.224
36.621
35.366
34.705
33.784
32.419
31.729
29.644
28.217
26.548
22.529
210
200
190
180
170
160
150
140
130
120
110
100
90
80
70
60
50
40
30
20
ppm

Figure S6

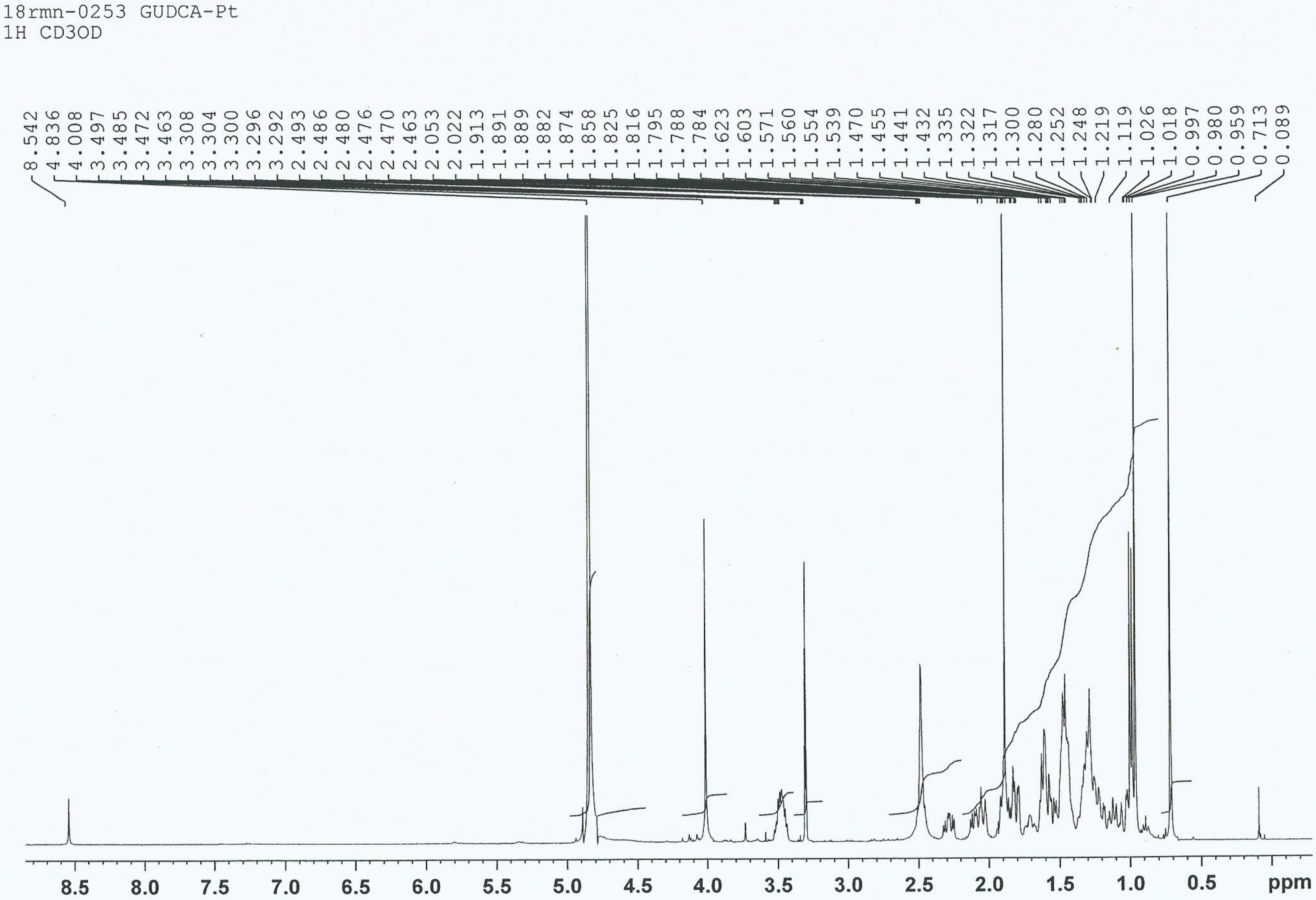
18rmn-0253 GUDCA-Pt
1H CD3OD
8.542
4.836
4.008
3.497
3.485
3.472
3.463
3.308
3.304
3.300
3.296
3.292
2.493
2.486
2.480
2.476
2.470
2.463
2.053
2.022
1.913
1.891
1.889
1.882
1.874
1.858
1.825
1.816
1.795
1.788
1.784
1.623
1.603
1.571
1.560
1.554
1.539
1.470
1.455
1.441
1.432
1.335
1.322
1.317
1.300
1.280
1.252
1.248
1.219
1.119
1.026
1.018
0.997
0.980
0.959
0.713
0.089
8.5
8.0
7.5
7.0
6.5
6.0
5.5
5.0
4.5
4.0
3.5
3.0
2.5
2.0
1.5
1.0
0.5
ppm

Figure S7

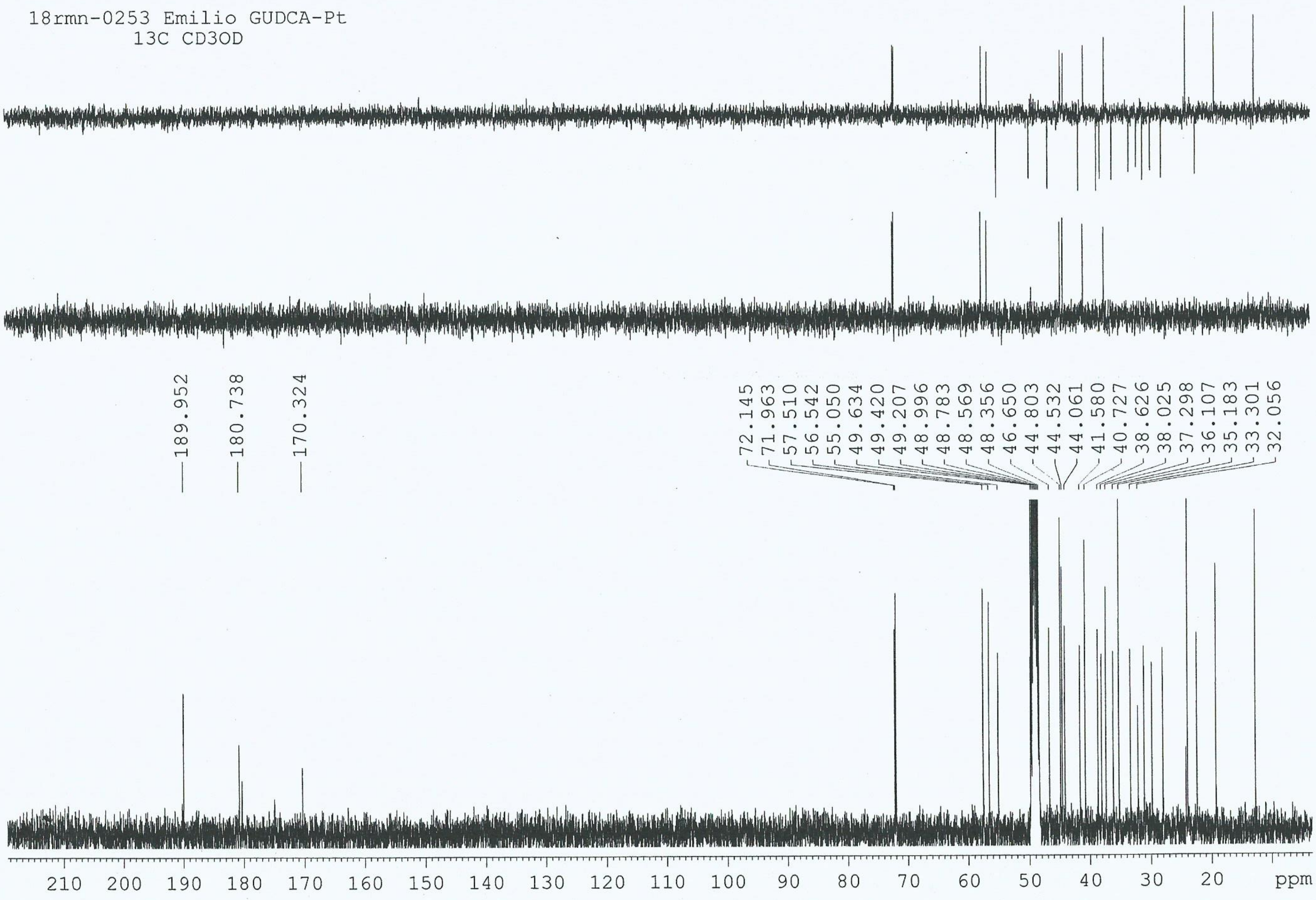
18rmn-0253 Emilio GUDCA-Pt
13C CD3OD
189.952
180.738
170.324
72.145
71.963
57.510
56.542
55.050
49.634
49.420
49.207
48.996
48.783
48.569
48.356
46.650
44.803
44.532
44.061
41.580
40.727
38.626
38.025
37.298
36.107
35.183
33.301
32.056
210 200 190 180 170 160 150 140 130 120 110 100 90 80 70 60 50 40 30 20 ppm

Figure S8

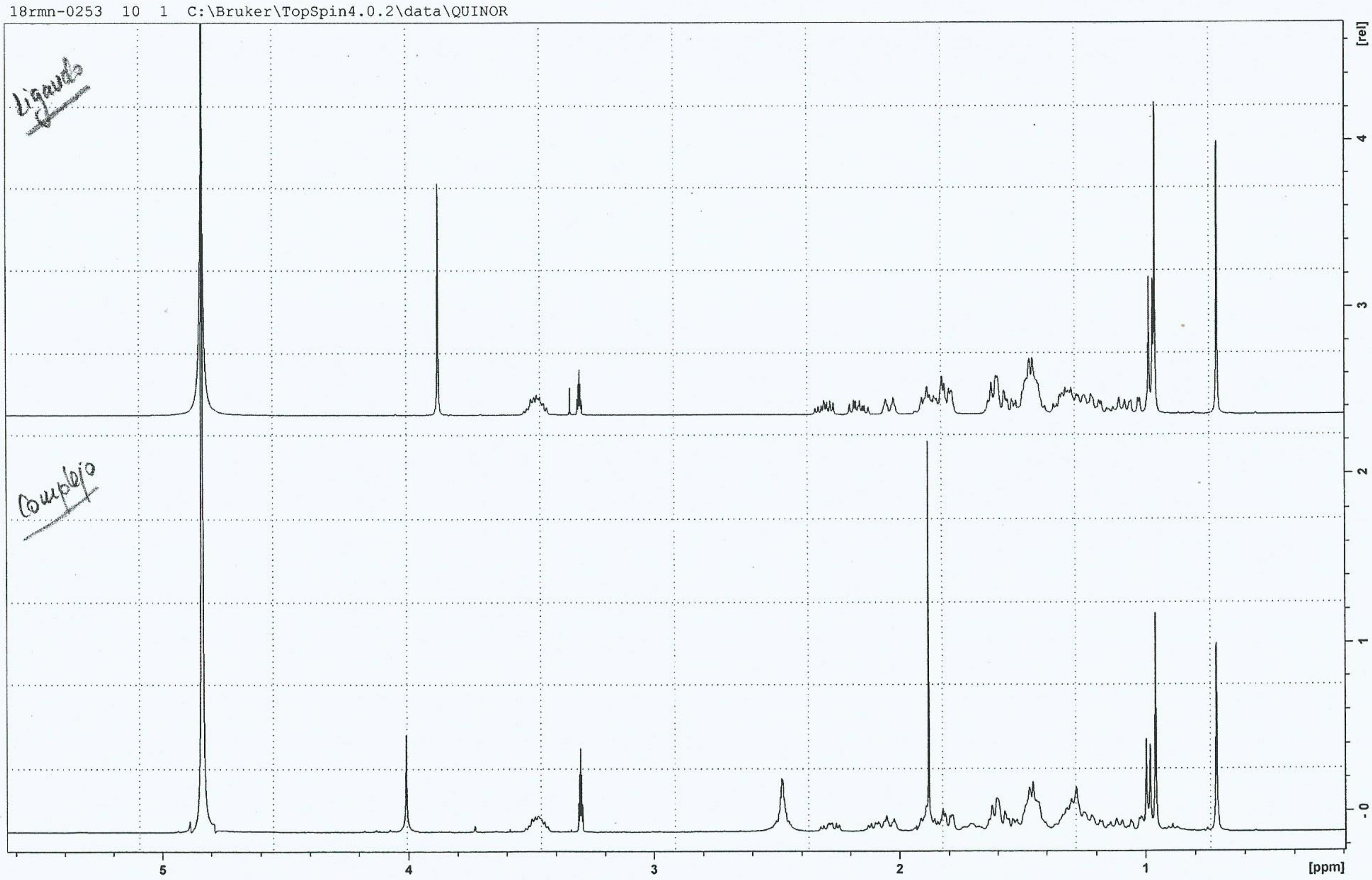

18rmn-0253 10 1 C:\Bruker\TopSpin4.0.2\data\QUINOR
Ligando
Complejo
[rel]
4
3
2
1
0
5
4
3
2
1
[ppm]

Figure S9

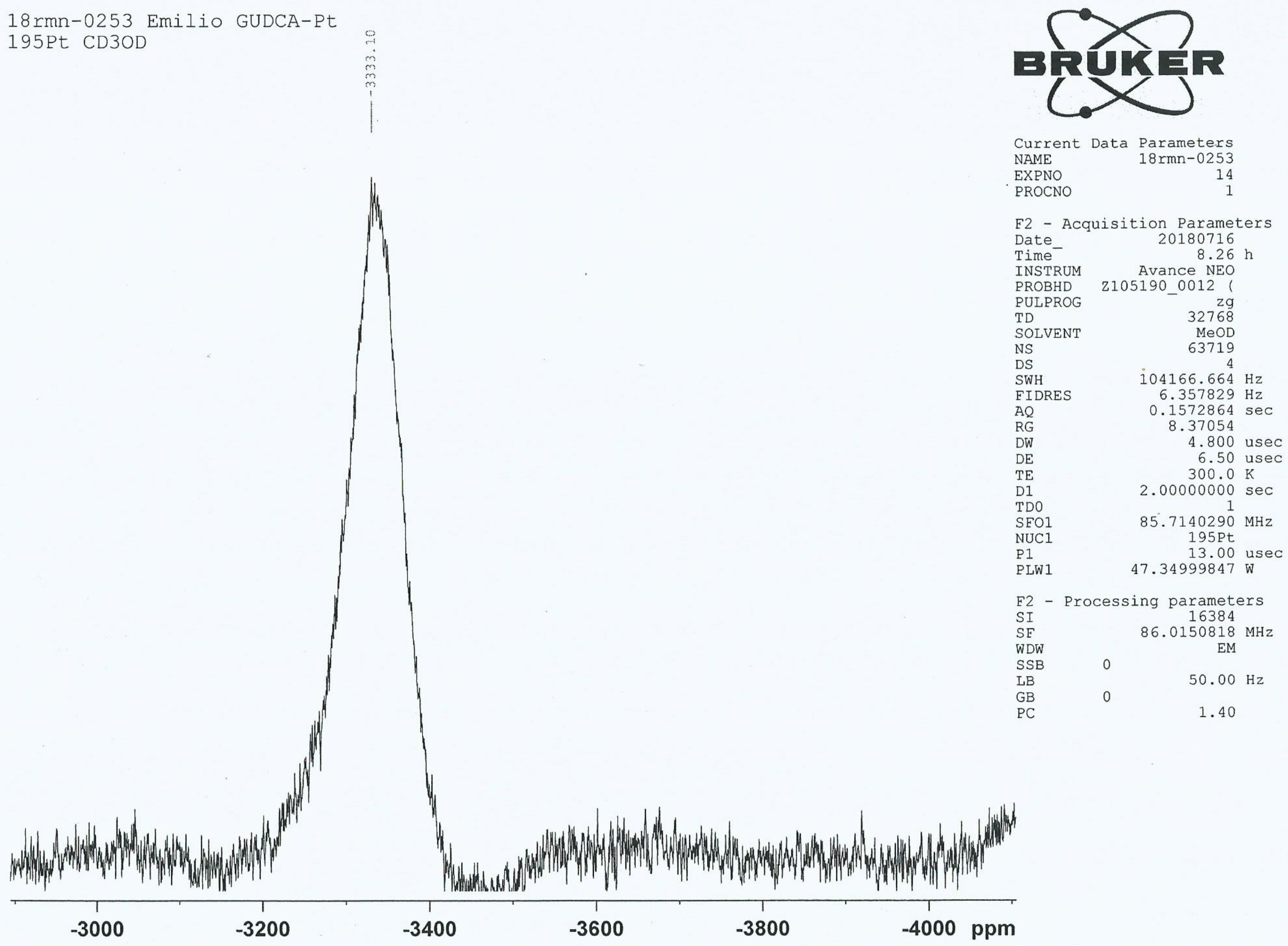

18rmn-0253 Emilio GUDCA-Pt
195Pt CD3OD
-3333.10
BRUKER
Current Data Parameters
NAME 18rmn-0253
EXPNO 14
PROCNO 1
F2 - Acquisition Parameters
Date_ 20180716
Time 8.26 h
INSTRUM Avance NEO
PROBHD Z105190_0012 (
PULPROG zg
TD 32768
SOLVENT MeOD
NS 63719
DS 4
SWH 104166.664 Hz
FIDRES 6.357829 Hz
AQ 0.1572864 sec
RG 8.37054
DW 4.800 usec
DE 6.50 usec
TE 300.0 K
D1 2.00000000 sec
TD0 1
SFO1 85.7140290 MHz
NUC1 195Pt
P1 13.00 usec
PLW1 47.34999847 W
F2 - Processing parameters
SI 16384
SF 86.0150818 MHz
WDW EM
SSB 0
LB 50.00 Hz
GB 0
PC 1.40
-3000
-3200
-3400
-3600
-3800
-4000 ppm

Figure S10

18rmn-0253 Pt-GUDCA
195 Pt CD3OD

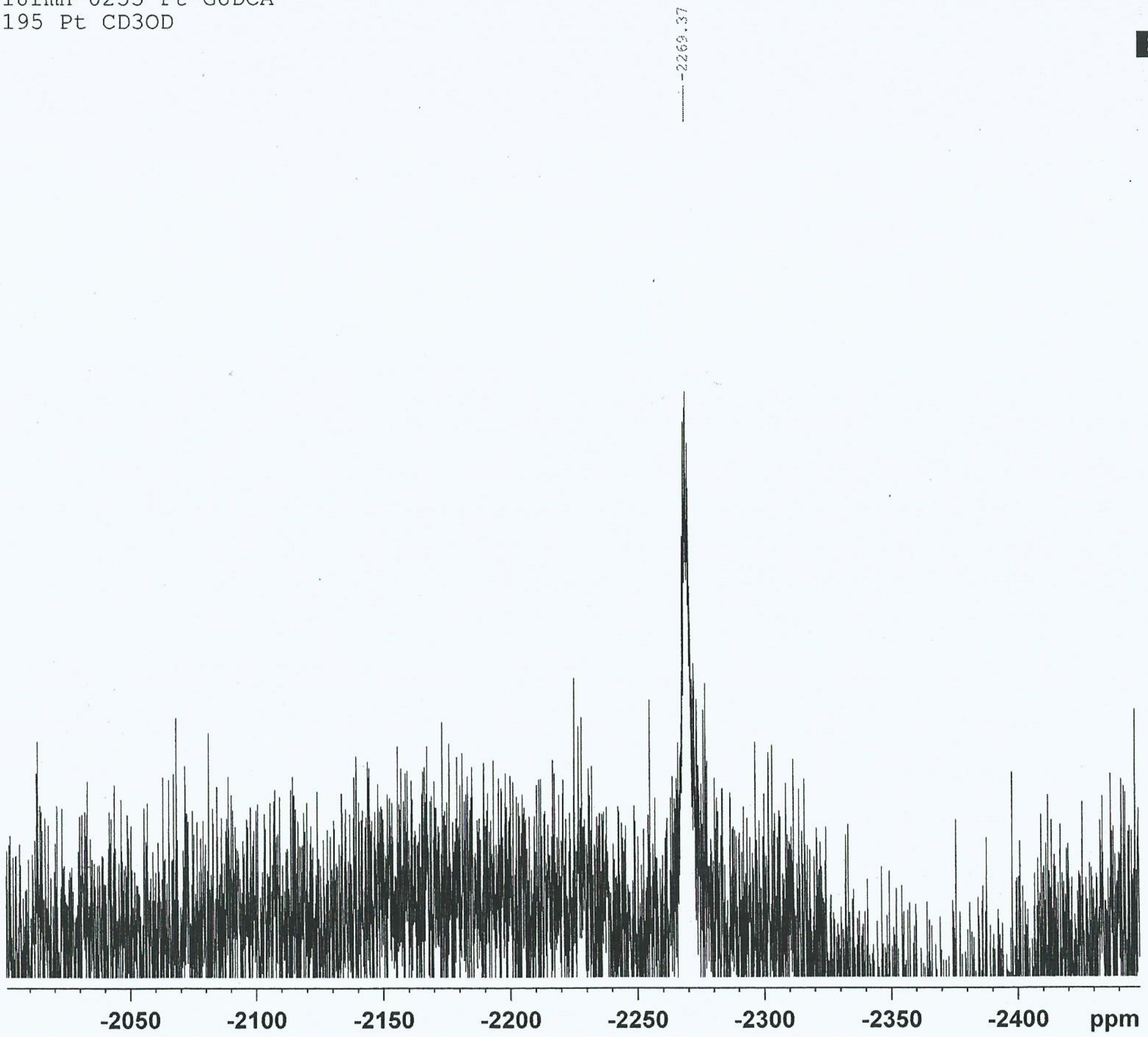


BRUKER

Current Data Parameters
NAME 18rmn-0253
EXPNO 15
PROCNO 1

F2 - Acquisition Parameters
Date_ 20180717
Time 7.05 h
INSTRUM Avance NEO
PROBHD Z105190_0012 (
PULPROG zg30
TD 32768
SOLVENT MeOD
NS 32768
DS 4
SWH 104166.664 Hz
FIDRES 6.357829 Hz
AQ 0.1572864 sec
RG 14.6484
DW 4.800 usec
DE 6.50 usec
TE 300.0 K
D1 2.00000000 sec
TD0 1
SFO1 85.8430517 MHz
NUC1 195Pt
P1 13.00 usec
PLW1 47.34999847 W

F2 - Processing parameters
SI 16384
SF 86.0150818 MHz
WDW EM
SSB 0
LB 50.00 Hz
GB 0
PC 1.40

Figure S11

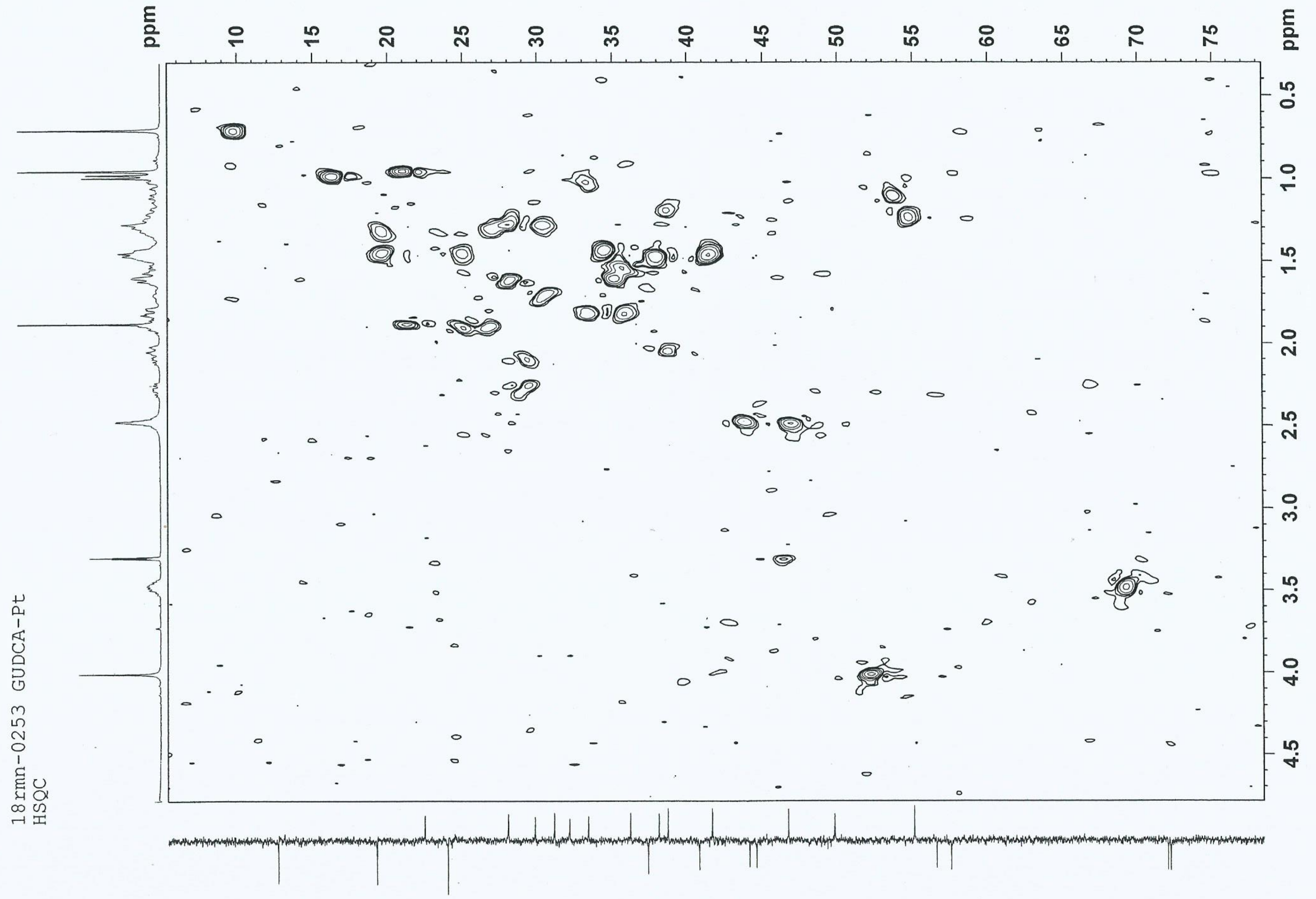
18rmn-0253 GUDCA-Pt
HSQC
ppm
10
15
20
25
30
35
40
45
50
55
60
65
70
75
ppm
0.5
1.0
1.5
2.0
2.5
3.0
3.5
4.0
4.5

Figure S12

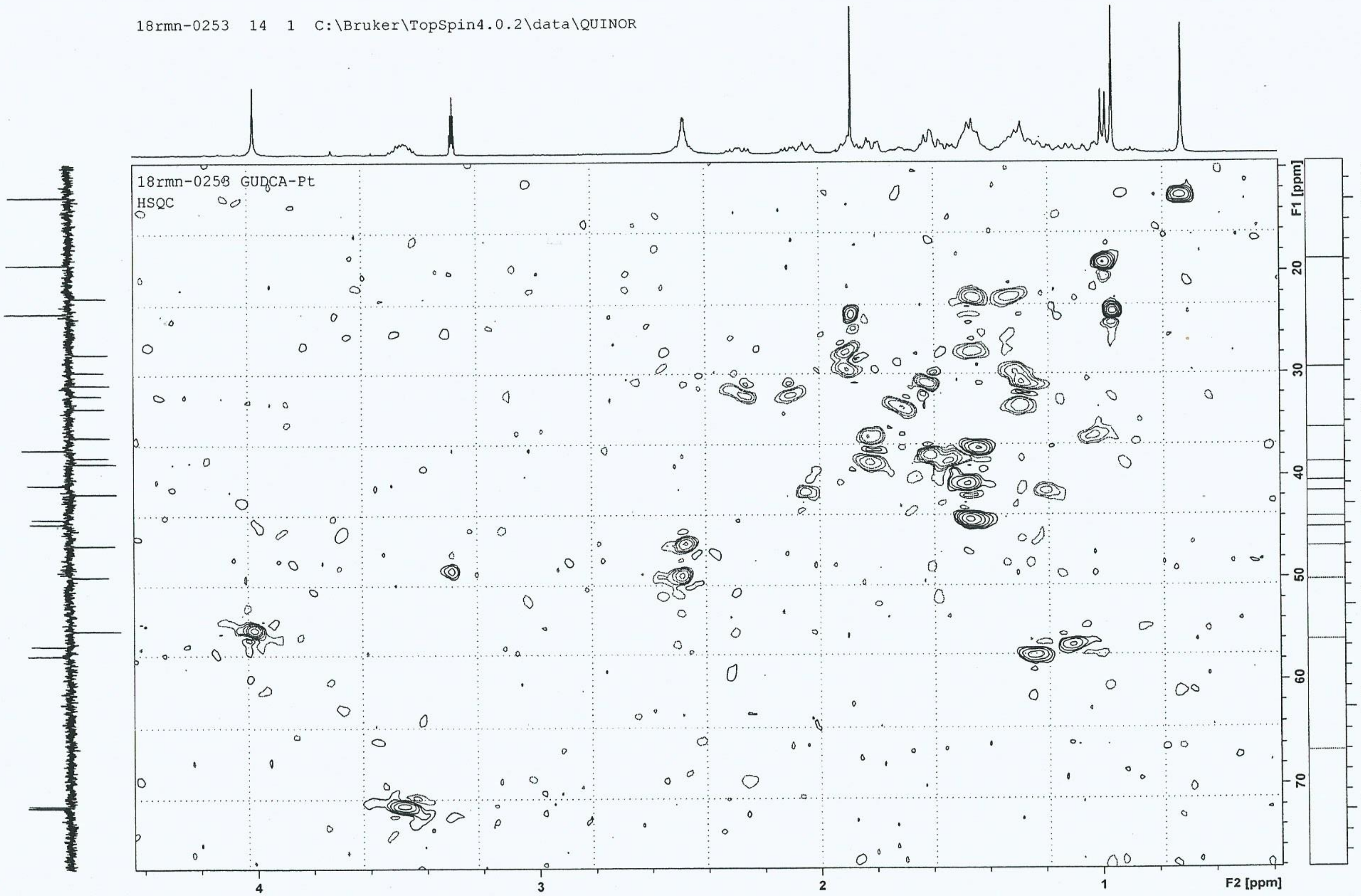

18rmn-0253 14 1 C:\Bruker\TopSpin4.0.2\data\QUINOR
18rmn-0253 GUDCA-Pt
HSQC
F1 [ppm]
F2 [ppm]
[ *1e9]

Figure S13

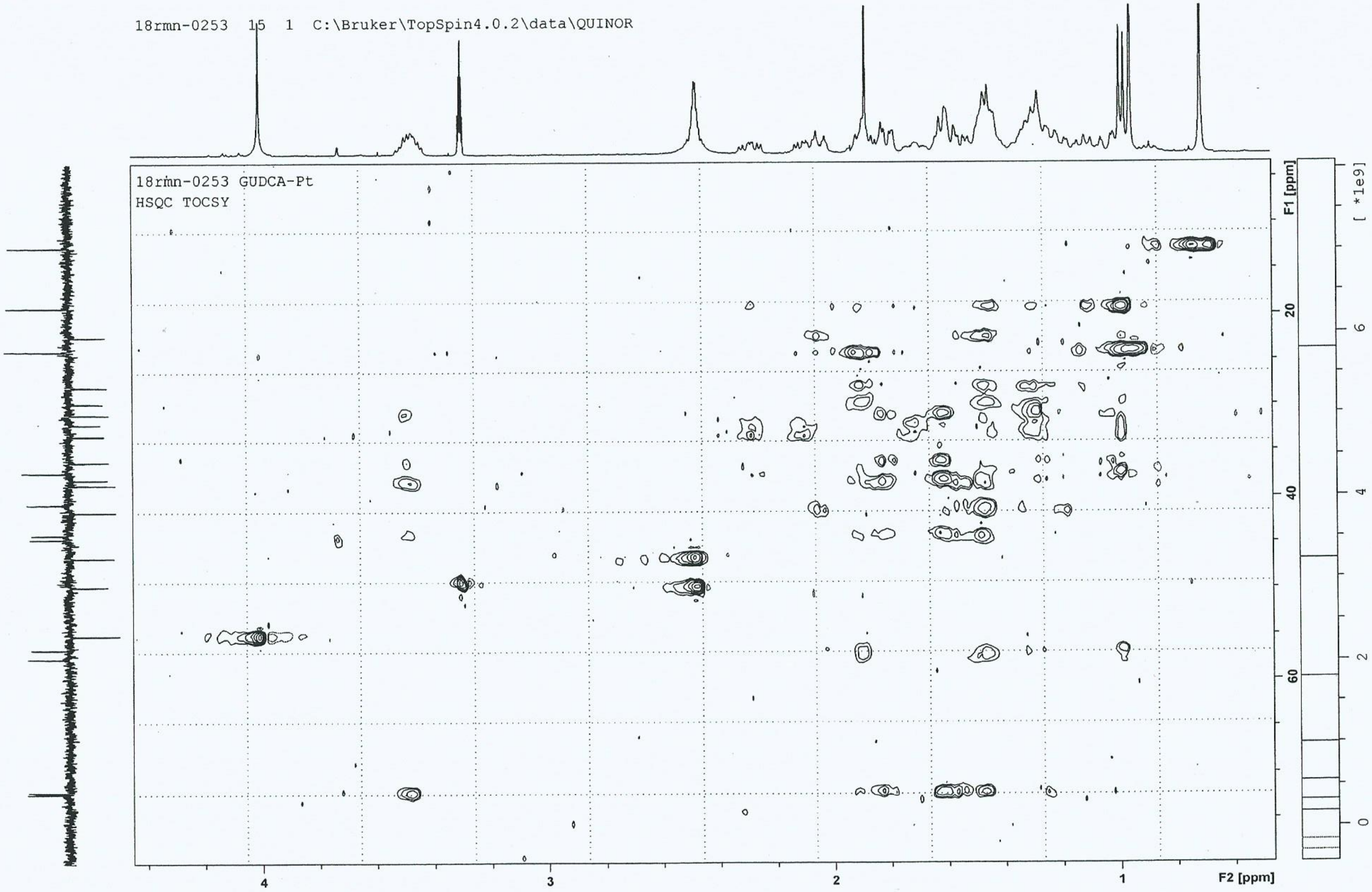
18rmn-0253 15 1 C:\Bruker\TopSpin4.0.2\data\QUINOR
18rmn-0253 GUDCA-Pt
HSQC TOCSY
F1 [ppm]
20
40
60
[ *1e9]
6
4
2
0
4
3
2
1
F2 [ppm]

Figure S14

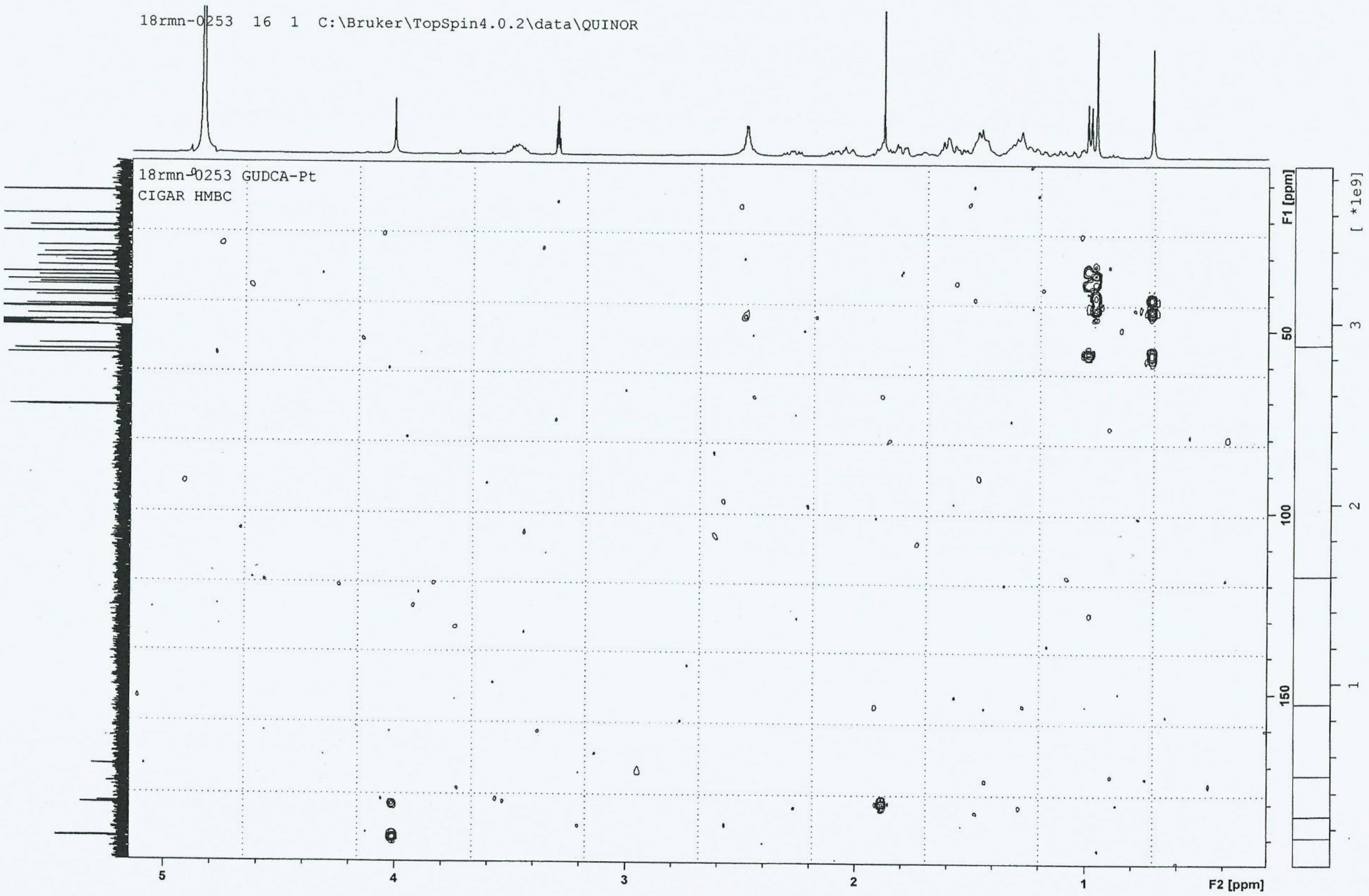

18rmn-0253 16 1 C:\Bruker\TopSpin4.0.2\data\QUINOR
18rmn-0253 GUDCA-Pt
CIGAR HMBC
F1 [ppm]
50
100
150
[ *1e9]
3
2
1
5
4
3
2
1
F2 [ppm]

Figure S15

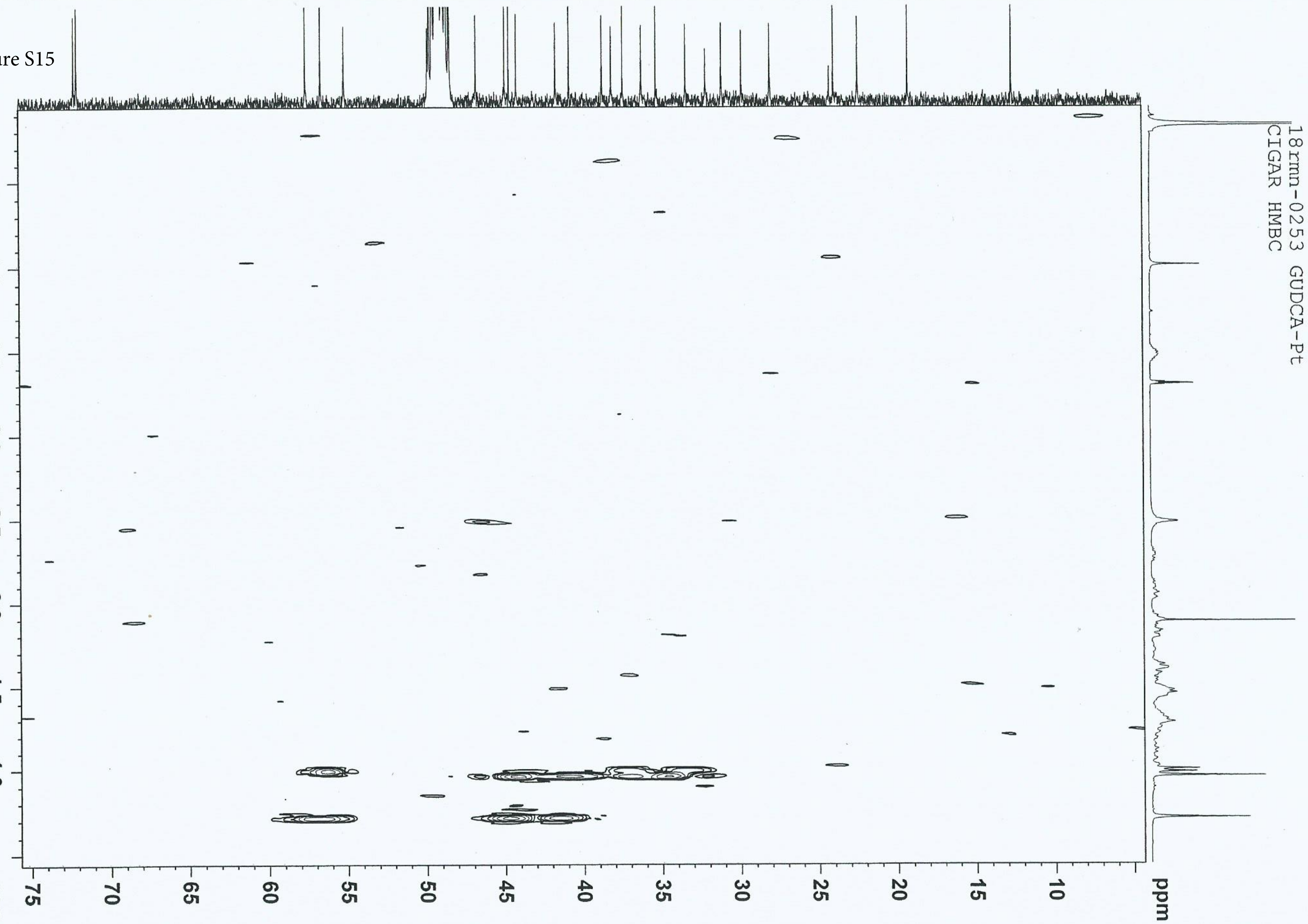

Figure S16

**SERVICIO DE ANÁLISIS ELEMENTAL, CROMATOGRAFÍA Y MASAS**
C/ Espejo s/n Edificio Multiusos I+D+i
Salamanca 37007

S. G. Espectrometría de Masas - USAL.
Solicitante: Emilio Rodriguez — Masa nominal
Emitido por: César Raposo (Responsable SGEM) — GU-Pt3

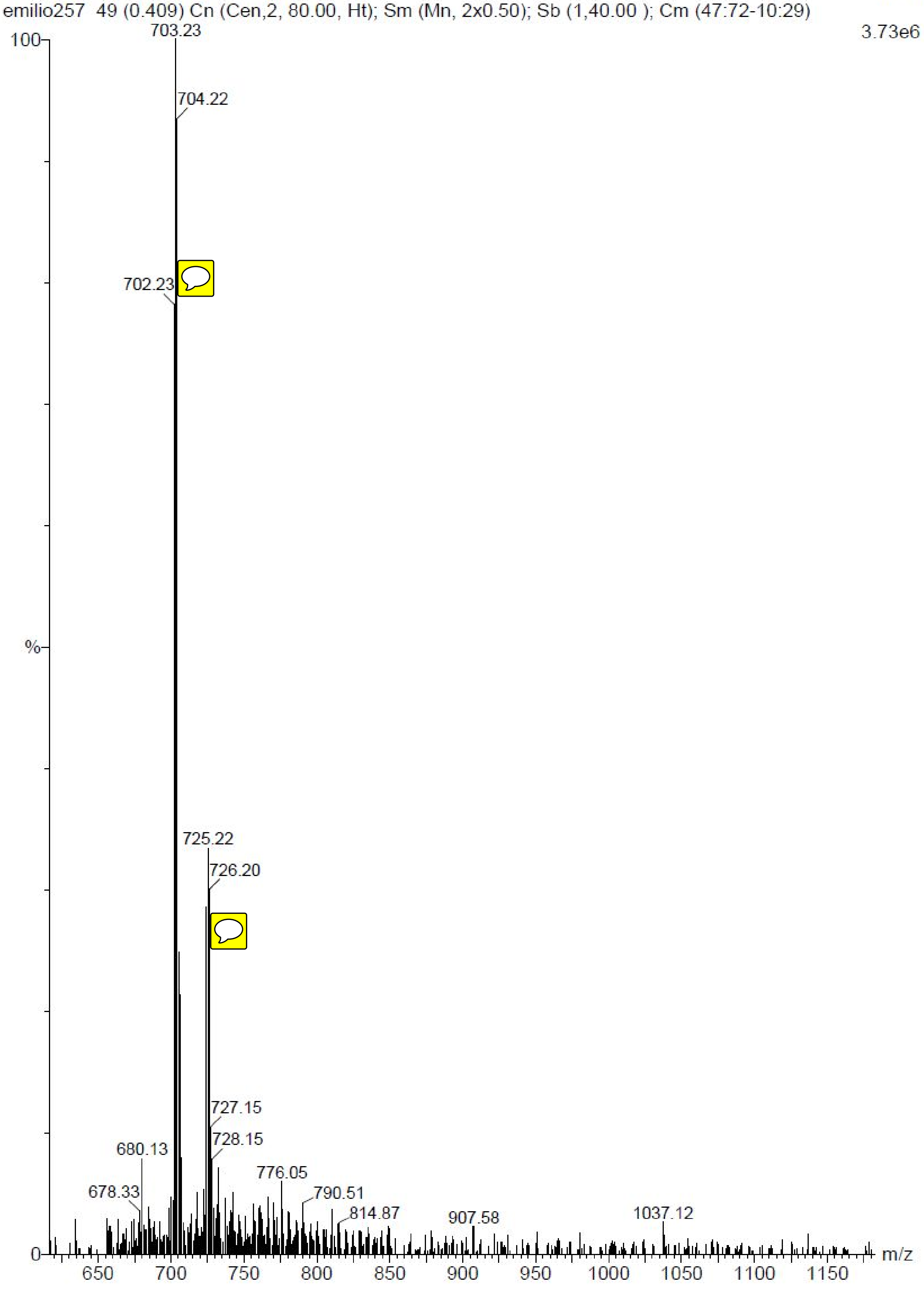

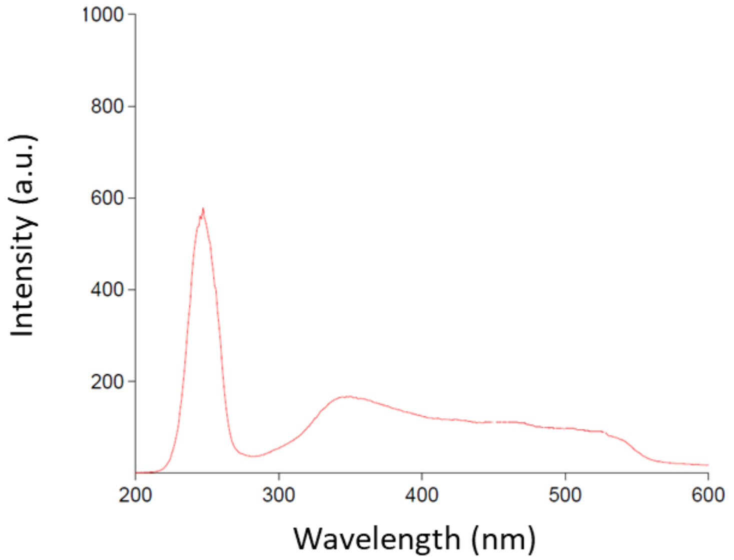


Figure S17: Fluorescence spectrum in water of [PtCl(GUDCA)en] ($4.33x10^{-4}$ M), λexc. = 240 nm. Quantum yield, Q = 0.10 (by comparison with quinine sulfate).

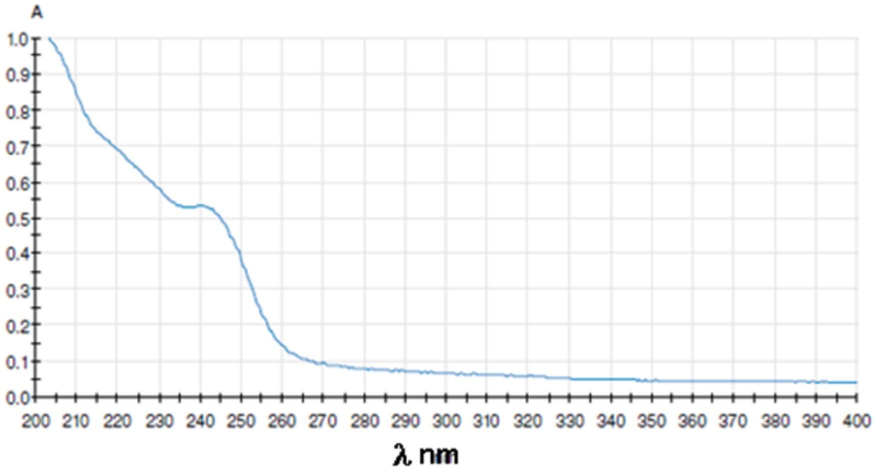


Figure S18: UV spectrum in water of [PtCl(GUDCA)en] ($6.37x10^{-6}$ M)

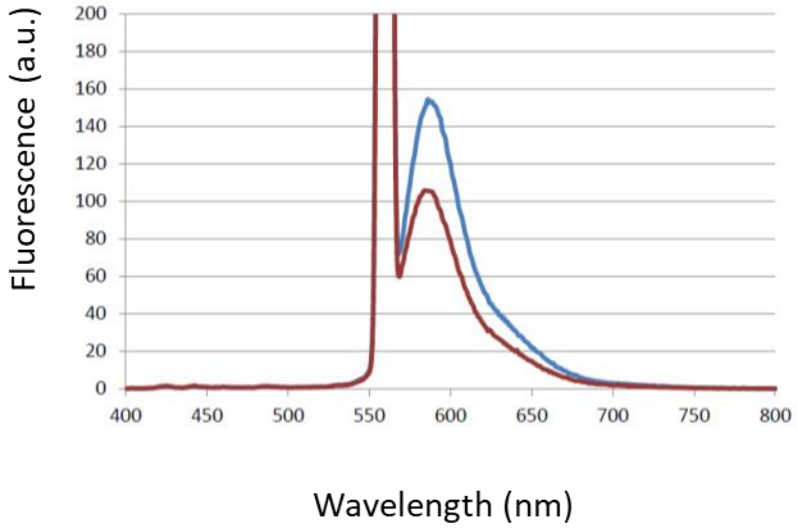


Figure S19: Fluorescence of IONP@BCP (C($Fe_2O_3$) = 0.097 mg/mL, brown) and IONP@BCP@[PtCl(GUDCA)en] (C(Pt) = 4.95x$10^{-4}$ M, blue). λexc. = 554 nm, λem. = 586 nm.

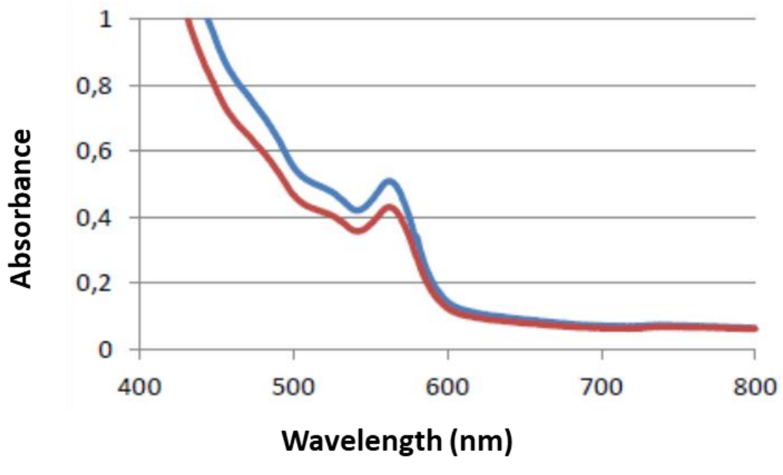


Figure S20: UV-vis spectra of IONP@BCP (C($Fe_2O_3$) = 0.097 mg/mL, brown) and IONP@BCP@[PtCl(GUDCA)en] (C(Pt) = 4.95x$10^{-4}$ M, blue).

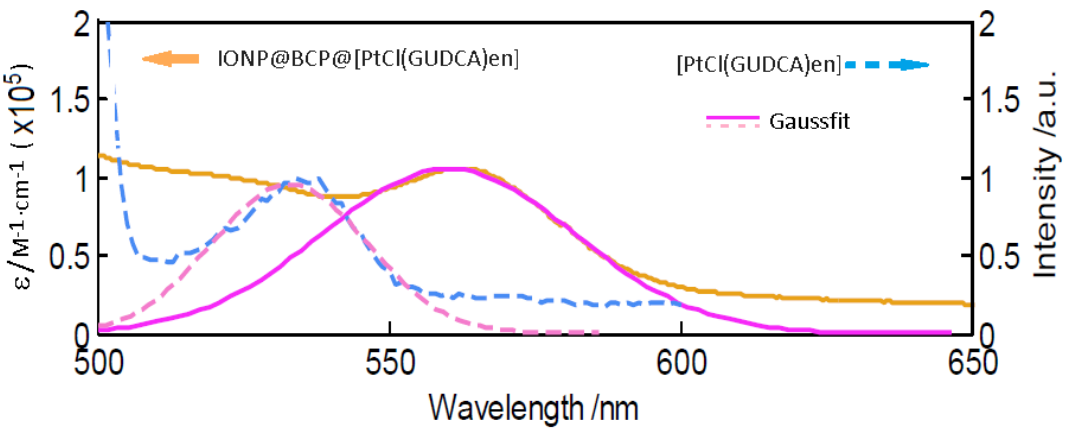


Figure S21: Normalized emission spectrum of [PtCl(GUDCA)en] and absorption spectrum of IONP@BCP@[PtCl(GUDCA)en]. The overlap integral (J) expresses the degree of spectral overlap between the donor emission and the acceptor absorption. In this case: J = 4.296x1015 nm4/(M·cm).

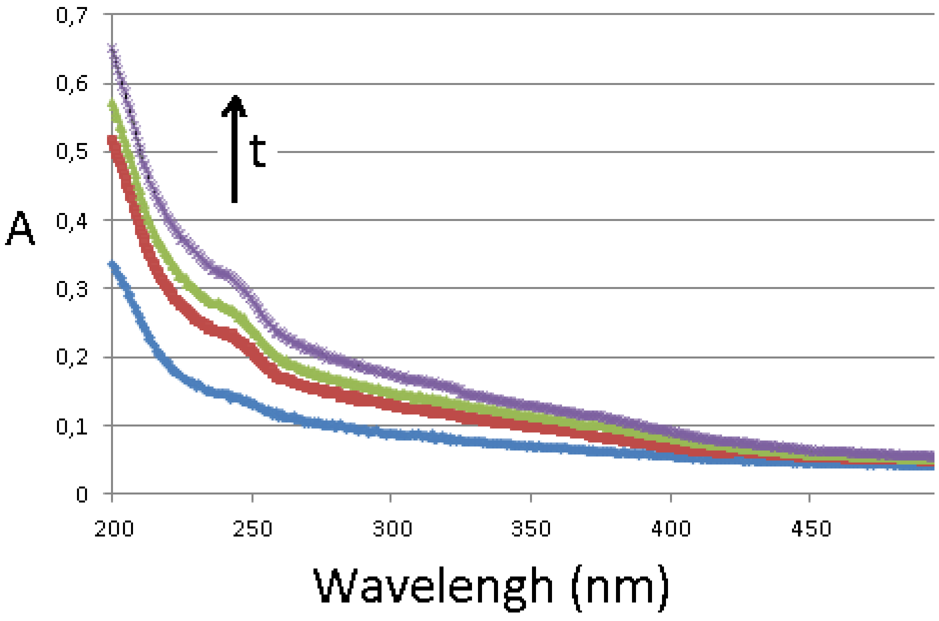


Figure S22: UV spectrum of Pt release versus time showing the characteristic shoulder of [PtCl(GUDCA)en] at 240 nm.

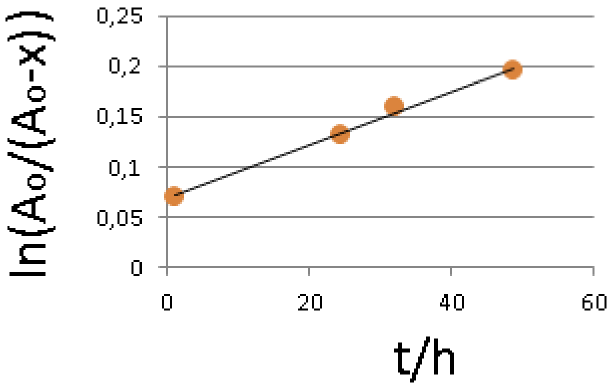


Figure S23: Representation of the data according to first-order kinetics, ln(Ao/(Ao-x)) = kt. Ao is the absorbance of the complex bound to the nanoparticles at t = 0; x is the absorbance of the complex released at time t in hours.

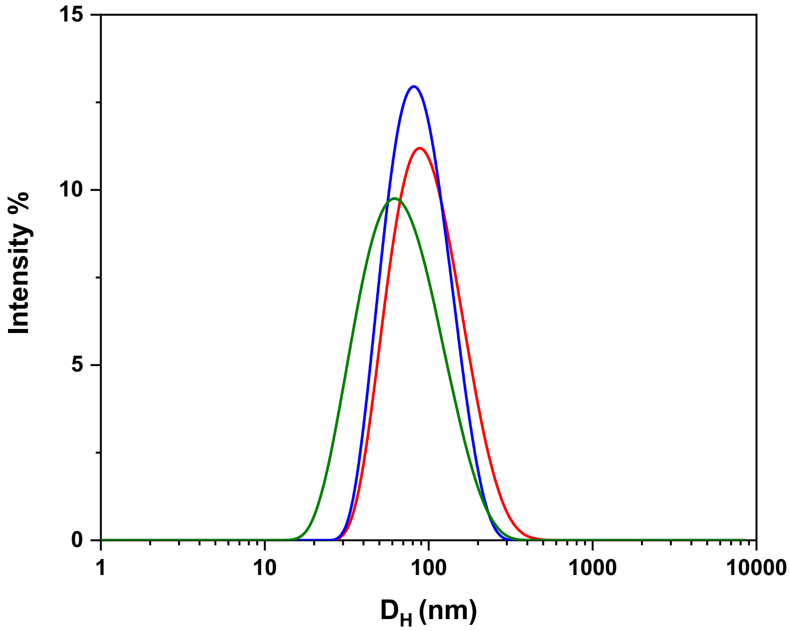


**Figure S24**. Intensity distribution of hydrodynamic diameters of NPs before coating (green), after coating (blue), and after functionalization with [Pt (GUDCA)$_2$en complexes (red).

**Table S1.** Intensity mean hydrodynamic diameter (DH) and standard deviation (SD) of the nanoparticle suspensions obtained from dynamic light scattering (DLS) measurements.

| Sample | DH (nm) | SD (nm) |
|---|---|---|
| IONP | 75.9 | 43.0 |
| IONP@BCP | 89.9 | 38.1 |
| IONP@BCP@ [Pt(GUDCA)en] | 107.5 | 54.1 |

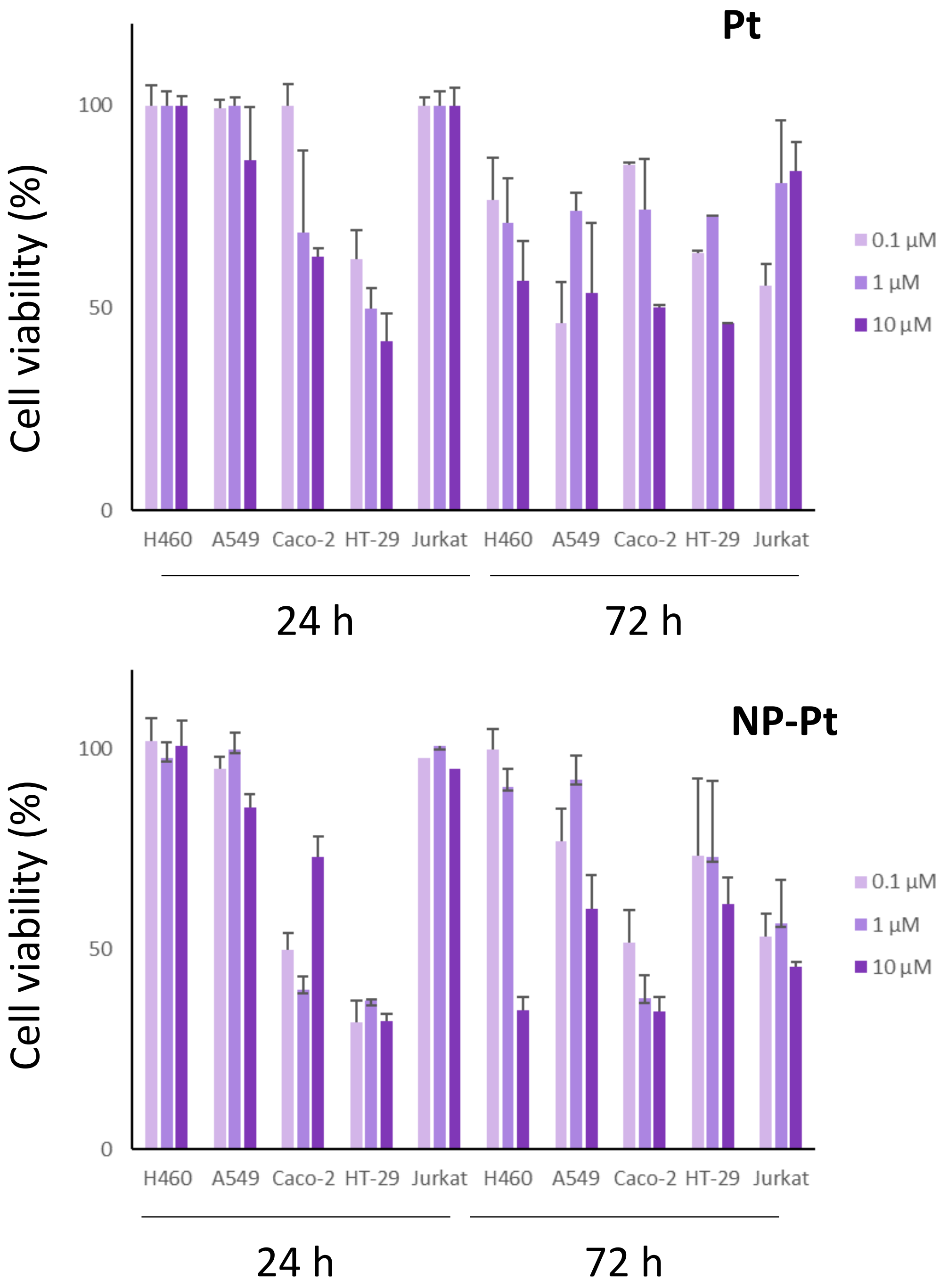


**Figure S25**: viability resultsof Pt compound and NP-Pt at 24 and 72 h of incubation in diferent concentrations (0.1, 1 and 10 uM).
