# Supplementary material for: Enhancement of Tumor Cell Immunogenicity and Antitumor Properties Derived from Platinum-Conjugated Iron Nanoparticles
Source: Cancers (Basel). 2023 Jun 15;15(12):3204. doi: 10.3390/cancers15123204 (PMC10296818; doi:10.3390/cancers15123204)
Supplement: Supplementary file 1 [file cancers-15-03204-s001.zip › HernandezAP_Cancers_SuppFile3.pdf]

Data analysis after processing the GenePix files

Each protein analyte was evaluated in 2 biological replicates each with 3 technical replicates. Negative fluorescence signal was transformed to zero. As expected, the signal distribution was not normal and zero-inflated (DENSITY PLOTS BELLOW). Therefore, non-parametric Man-Whitney test was used to compare the protein relative abundance between groups (experiment 1 or 2 Vs. Control).

Standardized effect size (Ratio) was calculated by subtracting the mean log2 fluorescence in condition 1 or 2 and mean log2 fluorescence in control and then dividing by the overall standard deviation of experiment and control.

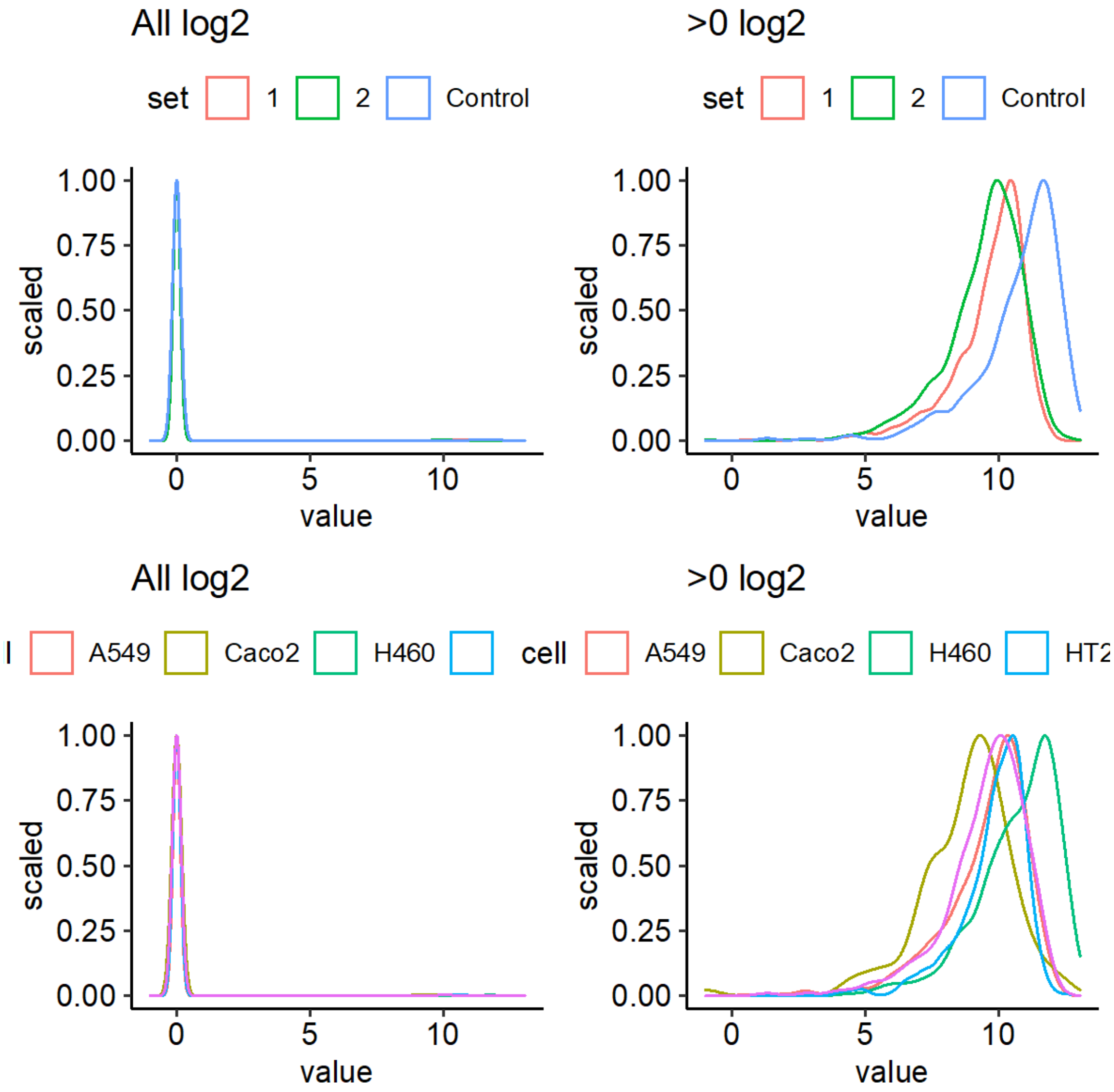

Ratio = Mean Condition X – Mean Control / SD (condition X and control)
Proteins with ratio > absolute(0.8) are transformed to -0.8 or 0.8 to fit in the scale
The order in Scale-X is the same in all the plots. Based in the rank ratio of H460 condition 2 Vs. control (red)

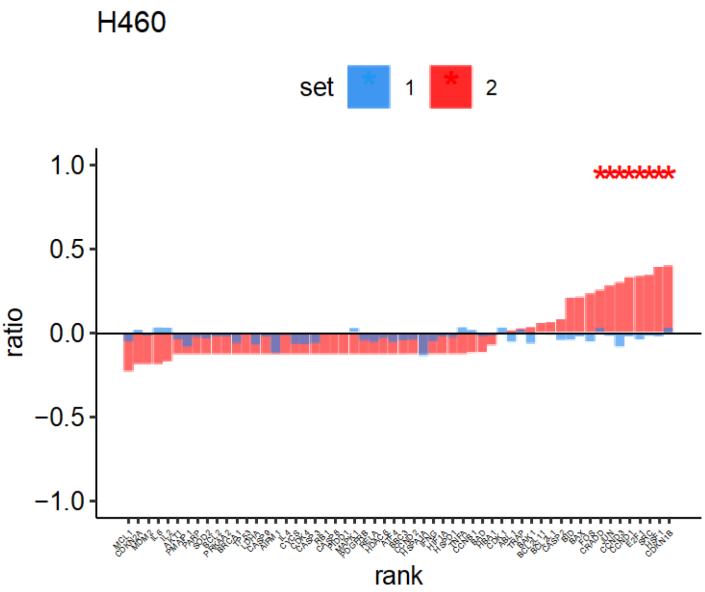

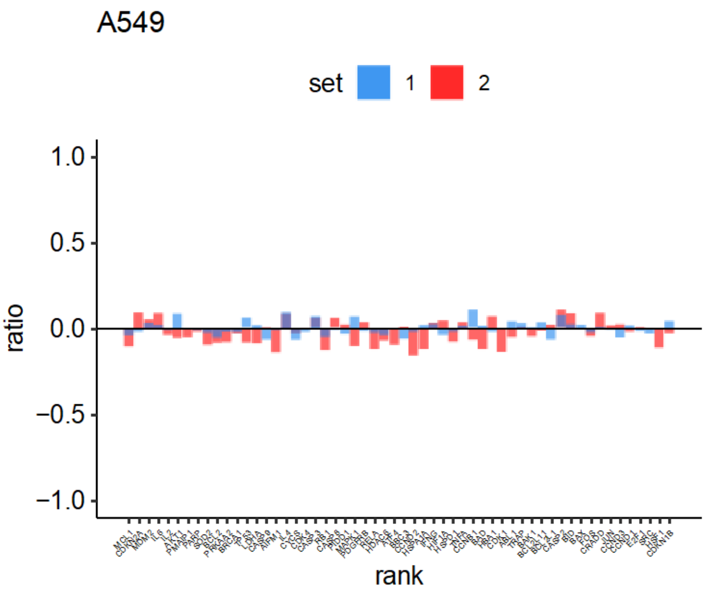

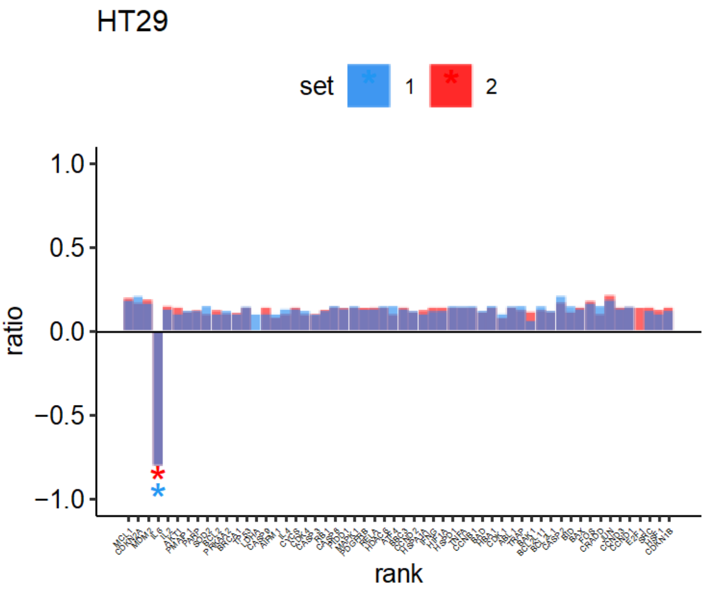

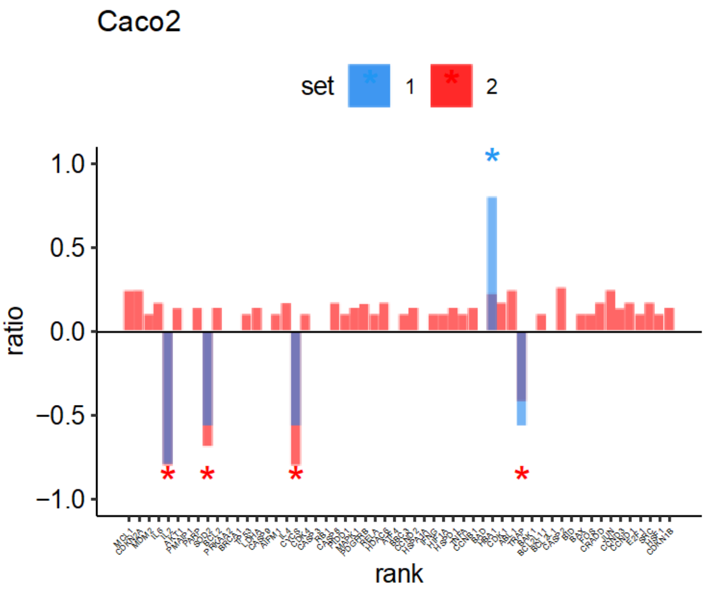

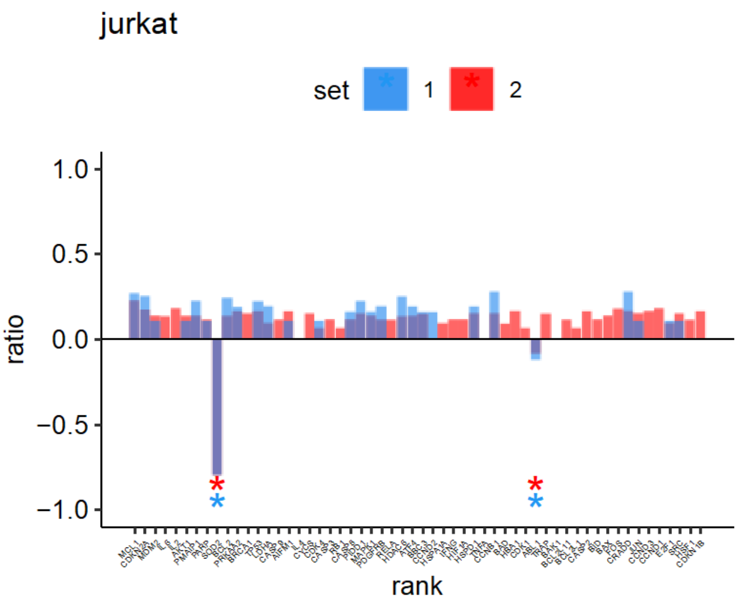

NP + Cis-Pt

Only Cis-Pt
